# Urine stabilization and normalization strategies favor unbiased analysis of urinary EV content

**DOI:** 10.1038/s41598-022-22577-3

**Published:** 2022-10-21

**Authors:** Riccardo Vago, Giorgia Radano, Davide Zocco, Natasa Zarovni

**Affiliations:** 1 Urological Research Institute, Division of Experimental Oncology, IRCCS San Raffaele Scientific Institute, Milan, Italy; 2 Università Vita-Salute San Raffaele, 20132 Milan, Italy; 3 Exosomics S.p.A, 53100 Siena, Italy; 4 HansaBiomed Life Sciences OU, Tallinn, Estonia

**Keywords:** Diagnostic markers, Translational research

## Abstract

Urine features an ideal source of non-invasive diagnostic markers. Some intrinsic and methodological issues still pose barriers to its full potential as liquid biopsy substrate. Unlike blood, urine concentration varies with nutrition, hydration and environmental factors. Urine is enriched with EVs from urinary-genital tract, while its conservation, purification and normalization can introduce bias in analysis of EV subsets in inter-and intra-individual comparisons. The present study evaluated the methods that decrease such biases such as appropriate and feasible urine storage, optimal single-step EV purification method for recovery of proteins and RNAs from small urine volumes and a normalization method for quantitative analysis of urine EV RNAs. Ultracentrifugation, chemical precipitation and immuno-affinity were used to isolate EVs from healthy donors’ urine that was stored frozen or at room temperature for up to 6 months. Multiple urine biochemical and EV parameters, including particle count and protein content, were compared across urine samples. To this purpose nanoparticle tracking analysis (NTA) and protein assessment by BCA, ELISA and WB assays were performed. These measurements were correlated with relative abundances of selected EV mRNAs and miRNAs assessed by RT-PCR and ranked for the ability to reflect and correct for EV content variations in longitudinal urine samples. All purification methods enabled recovery and downstream analysis of EVs from as few as 1 ml of urine. Our findings highlight long term stability of EV RNAs upon urine storage at RT as well as excellent correlation of EV content in urine with some routinely measured biochemical features, such as total urine protein and albumin, but not creatinine most conventionally used for urine normalization. Comparative evaluation of mRNA and miRNAs in EV isolates revealed specific RNAs, in particular RNY4 and small miRNA panel, levels of which well reflected the inter-sample EV variation and therefore useful as possible post-analytical normalizers of EV RNA content. We describe some realistic urine processing and normalization solutions for unbiased readout of EV biomarker studies and routine clinical sampling and diagnostics providing the input for design of larger validation studies employing urine EVs as biomarkers for particular conditions and diseases.

## Introduction

Urine is appealing and amenable source of potentially valuable diagnostic markers for a set of clinical conditions, most prominently those affecting the urogenital tract such as kidney diseases or injury, and urogenital cancers. In addition, urinary biomarkers have shown potential also for non-urological pathologies^1,2^ such as other cancers (i.e. Breast or Lung Cancer), metabolic and endocrine disorders (i.e. Diabetes), inflammatory conditions (i.e. atherosclerosis and osteoarthritis) and even neurodegenerative or neuropsychiatric diseases. The use of urine tests to diagnose diseases is an ancient practice (e.g. detection of glucose in diabetic subjects by tasting it or attracting ants, or detection of albumin as indicator of a renal disease by a “foam test”) and has remained an underlying component of investigative medicine throughout the 20th and the early twenty-first century^3^. With the advent of modern -omics techniques, a myriad of urine components is revealed whose quantitative and qualitative alterations are expected to have diagnostic and predictive value in the clinical arena.

Paradoxically, urine is still understudied as a totally non-invasive source of liquid biopsy biomarkers that can be used longitudinally for diagnosis and monitoring. This paradox is due to particular features of this biofluid, and to the methodological hurdles still interfering with the passage of biomarker candidates to the quantitative prerogative of validation and diagnostic assay implementation.

Unlike blood whose composition and volume are tightly regulated, urine is not subjected to homeostatic mechanisms. Urine is a unique biofluid that can range in pH, osmolality, composition and concentration of dispersed solutes, even within the same individual and over hours or days^4^. Urine production in adult humans varies within a normal range of 0.6–2.6 l. These huge inter-and intra-individual fluctuations in water excretions and in urine concentration that depend on diet, hydration, activity or environmental factors, impede objective measurements that are critically defining a biomarker as such. Reliable identification and evaluation of urinary biomarkers is likely to benefit from robust and specific platforms that promise to better define the origin (in terms of tissue or disease specificity) of biomarkers, in either soluble or solid urine phase. The letter comprises sediment (typically shed cells) and extracellular vesicles (EVs).

EVs hold promise as carriers of cell-specific traits, that encapsulate, and thus stabilize, rare but informative biomarker species (proteins, glycans, nucleic acids, lipids, metabolites)^5^. In such a way they act as real-time sentinels of physiological or pathological tissue alterations. Early scientific advances in investigating the urinary EV biomarker potential have focused mainly on urogenital cancers and have later inspired the quest for urinary EV biomarkers for other urogenital tract pathologies^6^. Indeed, the first and only commercial grade EV-based diagnostic kit, an IntelliScore, that has been developed and launched in 2018 by Exosome Diagnostics, is based on assessment of a defined EV-mRNA panel in urine samples. The usefulness of this test for patient stratification into advanced vs indolent Prostate Cancer has been extensively validated in prospective multi-center studies^7,8^. and today the same investigators group is applying a similar concept for development and validation of a new urinary EV-RNA based noninvasive test for transplant rejection^9^. However, for a vast list of urinary EV candidate biomarkers portrayed over last years for different pathologies, no significant validation has been accomplished, with comparison among the studies remaining extremely challenging. It is a state-of-art consensus^6^ that some methodological aspects of EV separation and analysis, including the preanalitycal handling and normalization of results, still persist, and need to be optimized and standardized in order to foster translation of scientific findings into clinical practice.

While EVs are ubiquitously present in urine, their concentration is low relative to more abundant secreted proteins such as uromodulin. Indeed, uromodulin is found to be most abundant protein contaminating the EC preparations in proteomic studies. The degree of contamination depended on proband samples and EV purification method used. Though considered as technical challenge in urine EV extraction efficiency, uromodulin contamination did not show the obvious reduction in banding of other abundant EV proteins in shot gun proteomics, and did not interfere with the analysis of EV urinary RNA cargo^10^.

Urine EV content is potentially contributed by different tissues, with the expected enrichment with vesicles released from distinct segments of urogenital route^6^. Recent research has indicated that EV heterogeneity is even more pronounced than previously anticipated, with bioinformatic analysis of omics data showing a broad representation of molecules in urinary EVs from across the body, without expected huge bias toward kidney ore urine proteins^10^. Different isolation protocols for proteomic and transcriptomic analysis of urinary EV have been described, likely to isolate different subpopulations of vesicles or at least enable different yield and purity, each of which may be appropriate for different clinical questions^4,11^. We have focused on the methods that allow single-step EV isolation and are suitable for scalable volumes, allowing the processing and analysis of both routinely harvested, and biobanked urine samples. Sample conservation and processing impacts detection of urine contained analytes, including EV subsets, and the normalization approach can introduce bias in inter and intra-individual/population comparisons. We have addressed the storage methods that are applicable in normal clinical practice as well as facilitating the logistics of samples exchange in multicenter studies. Several methods have been proposed to-date for normalization of urinary biomarkers discovery including the time normalization, creatinine or protein normalization^12–15^. As EVs are affected by concentration huge concentration fluctuations typical for all urine components, the applicability of similar methods to EV biomarkers was proposed and often adopted without the comparative evaluation of different methods or correlation to the independent EV parameters within the same study. Time normalization, in particular cumulative -to- 24-h urine collection, is difficult to implement and would rule out biobanked samples that are typically comprising spot urine. From a practical point of view, spot (random) urine, or alternatively first/second morning void urine, are preferred as usually collected during the clinical consultations. Creatinine is most often used to calibrate urine measurements also in the studies that involve urinary EVs^16^, but its variations are not completely attributable simply to variations in urine concentration. For this reason, normalization to creatinine can mask disease correlated quantitative variations and flaw interpopulation comparisons or even longitudinal monitoring. More common and reliable normalization method suitable for EV markers deployment is needed.

In this study we have first addressed the conditions for urine storage and shipment as well as feasibility of purification method appropriate for low volumes (typical for biobanked samples) that grant reliable recovery and sufficient quantities of EV markers. In the second part of the study we investigated the normalization method including EV counts and creatinine/protein measurement that are to-date commonly used in reported studies on urinary EVs, and comparing them to urine biochemical indicators, and absolute and relative values of specific EV markers. Although we describe a pilot study based on a small set of proband urine samples, it served us to establish the correlation and ranking of different urine and EV parameters that can be addressed in larger validation studies, run in an independent cohort(s), and in combination with the specific condition biomarkers. Specification and integration of these fundamental requirements will ultimately contribute to a roadmap to urine EV biomarker validation and deployment.

## Materials and methods

### Study design

In the first part of the study, we performed a methodological comparison, encompassing 2 storage temperatures (− 80 °C and RT), at 2 time points (month and 6 months) and 3 methods used for urine EV extraction (differential ultracentrifugation (UC), immunoaffinity-based pull-down (IP), or chemical precipitation (CP). We used 3 proband donors (healthy donors, Caucasians, male, 35–45 years old). To decrease the impact of a random operational errors to a small sample size, we have independently processed two aliquots for each urine sample. In such a way we could (1) evaluate the standard deviation between technical replicates and (2) evaluate additional biological replicates for each sample and assay used. NTA, BCA and creatinine assessment was done also no neat urine (prior to EV purification). All these variables were combined into a comprehensive design that also included parallel testing of several independently measured urinary EV biophysical and biochemical parameters. In such a way, although starting from only 3 proband donors, we had a consistent number of experimental points to analyse (path 1A in the scheme below).

In the second part of the study, we addressed the issue of inter- and intra (day-to-day) sample variations in EV content, when we would not expect that the physio-pathological status of our healthy probands has drastically changed. We started from 2 proband samples, healthy donors, 1 female (Caucasian, 45 years) and 1 male (Caucasian, 43 years), of each three longitudinal samples have been collected. For each collected samples, 2 urine aliquots were independently processed by UC as a protocol that allowed us to assess and compare large number of EV parameters, as described (path 1B in the scheme below). Neat urine has been used for NTA, BCA, and complete biochemical urinalysis.

**Figure:**
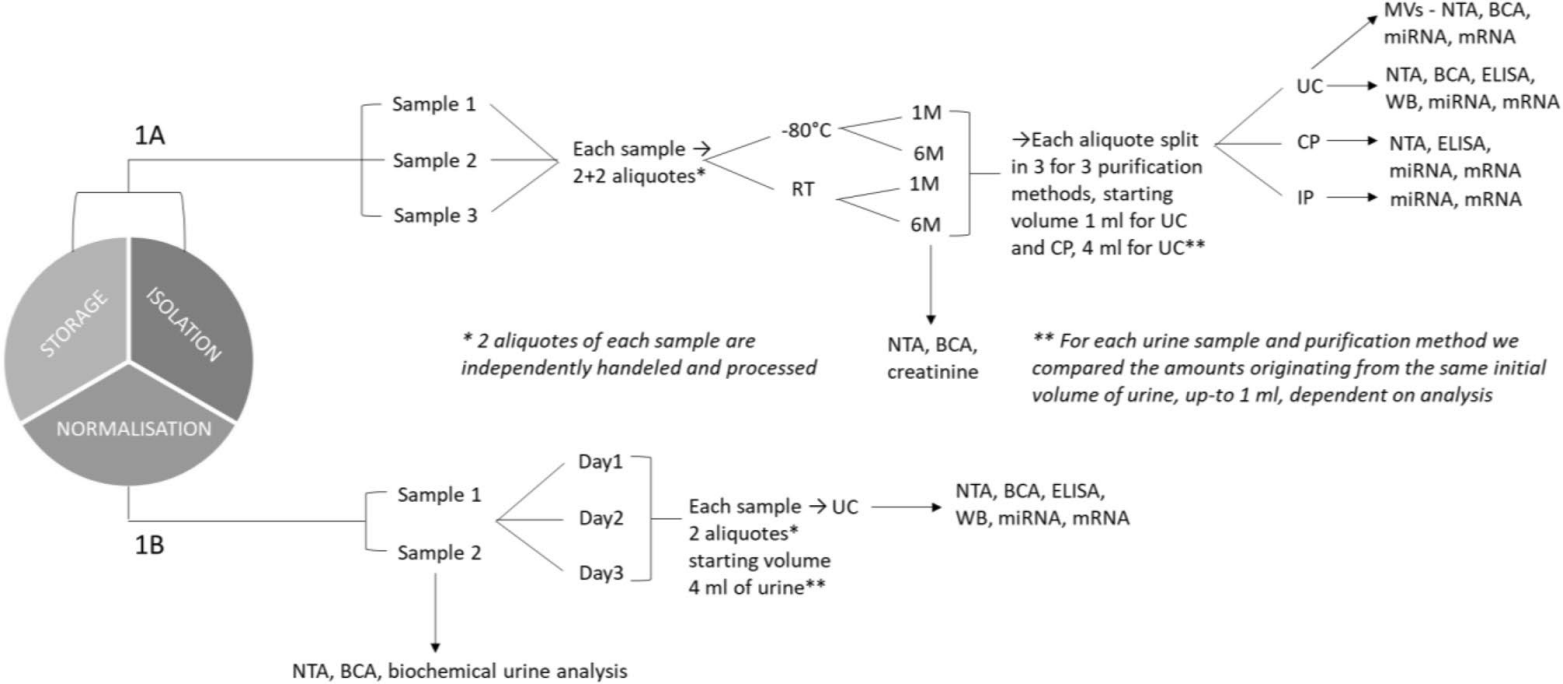
Schematic representation of the workflow carried out in the study.

### Urine harvesting and storage, biochemical analysis

First morning urine was collected from healthy volunteers (Caucasian, aged 43–45 years, see details in “Study design”) in a sterile screw-cap container, kept within 1 h at RT till processing (part B of the study) or storage (part A). Prior to further processing or storage, urine was spined at 300 g, at RT for 15 min (Eppendorf, Centrifuge 5804 R) to remove a gross precipitate (cells and debris). Urine is stored for 1 or 6 months either at − 80 °C, or supplemented with Urine Preservative (Norgen Biotek Corp.) according to manufacturer recommendations. In case of freezing, urine samples are first frozen at − 20 °C and shortly after placed at − 80 °C. This is compliant with the capacity of urine harvesting hospital practice. Once thawed, it was warmed to 37 °C and again spined at 300 g, prior to analysis and/or EV purification. The neat urine (prior to EV isolation) was used for Nanoparticle Tracking Analysis (NTA), protein content assessment (BCA) and ELISA, as well for comprehensive biochemical urine analysis performed at the clinical analysis laboratory of San Raffaele Hospital.

### Urine EV purification

After 1 or 6 months storage, and, in case of frozen samples—rapid thawing and brief warming up to 37 °C, we have first pre-cleared urine by 300 × g centrifugation to remove sediment and subsequently spin the sample at 12,000 × g to remove MV pellet. The supernatant was than subjected to 3 exosome purification protocols employing ultracentrifugation (UC), chemical precipitation (CP) and immunobeads (IP) mediated pull down. Starting urine volume was 4 ml for UC and 1 ml for IP and CP, while for subsequent protocol comparison the sample amount corresponding to equal original 1 ml of urine was considered. For each urine samples, two aliquots were independently processed with each purification method as to assess the robustness of methods used (see “Study design”). Warming up of the sample prior to sample pre-clearing rendered the urine limpid and kept abundant urine components such as uromodulin in solution, avoiding thus the loss of EVs due to an entrapment in large protein gel-like complexes during the low centrifugation steps. We have also confirmed normal to slightly alkaline pH value of used urine samples (7.5–8) using litmus paper measurement^1^. We preferred this procedure to other steps reported to alter the solubility of uromodulin such as DTT or detergent treatment^3,4^. Ultracentrifugation protocol was adjusted from Cheng et al.^17^. Briefly, urine was submitted to sequential centrifugation steps, comprising the low speed 15,000 g centrifugation for 10 min for collection of microvesicles (MV), and subsequent ultracentrifugation and washing steps at 110,000 g (Beckman Coulter, Optima L-90K Ultracentrifuge, fixed-angle rotor) for 2 h. All centrifugation steps are performed at RT (22 °C). Washing is done in particle-free 1×PBS (Gibco) and no additional filtering steps were applied to further exclude or select EVs by size. EV recovery by IP protocol was based on the use of commercially available NH2-latex beads (400 nm) (HansaBioMed Life Sciences, Tallin, Estonia) coated with anti-CD9 antibodies, following the manufacturer’s instructions and prior published protocols^18^. The samples were incubated with 10 μl of beads at 4 °C on a rotator. Upon EV binding, beads were recovered by top bench centrifugation (5000 × g for 10 min) and washed three times with PBS 0.05% Tween-20 (PBST). For EV isolation by CP, samples were treated with Exo-Prep precipitation reagent (HansaBioMed OU, Tallin, Estonia), according to the manufacturer’s instructions. Briefly, upon addition of the Exo-Prep reagent (vol. ratio 1:4), the samples were incubated for 1 h in ice. The exosome pellet was recovered by the bench centrifugation at 7000 × g, for 10 min, and washed three times in PBS.

### Nanoparticle tracking analysis

Nanoparticle tracking analysis (NTA) was performed using a NanoSight LM10-HS microscope (NanoSight, Ltd.) equipped with a 405 nm laser using embedded NTA software v3.00. Samples were diluted with PBS to reach a working concentration of 20–120 nanoparticles per frame and/or 10^7^–10^9^ nanoparticles/mL. For each sample, three 30-s videos with a frame rate of 30 frames per second were recorded. Temperature was monitored and recorded throughout the measurements. Captured videos were analyzed by NTA software (version 3.2) (Malvern Panalytical) to determine nanoparticle concentration and size with relative standard error. For analysis, automatic settings were used for blur, minimum track length, and minimum expected particle size. Prior to analysis, calibration of the NanoSight system was performed using polystyrene latex microbeads with a size of 100, 200 nm, and 400 nm (NanoSight, Ltd.).

### Protein content estimation

Measurements of protein concentration in all analyzed samples were performed using the Pierce™ BCA Protein Assay (Thermo Fisher Scientific), according to the manufacturer’s instructions.

### ELISA-based exosome immunocapture and quantification

EV-based sandwich ELISA assay (ExoTEST™; HansaBiomed Life Sciences) was performed in 96-well microplates, according to the manufacturer’s protocol. Briefly, microplates coated with primary capture antibodies anti-CD9 and anti-CD63 were incubated with standards and samples overnight at 4 °C. Microplate wells were treated with primary detection antibodies anti-CD9-bio, followed by incubation with HRP-Streptavidin-conjugated secondary antibodies and TMB Substrate Solution. Stop Solution (2 N H_2_SO_4_) was added to each microplate well. Assay was calibrated using a standard curve prepared with linear dilutions of exosomes purified from human urine (HBM-PEU; HansaBioMed Life Sciences) embedded in the ELISA kit as standards. Op-tical densities were determined using a microplate reader Infinite-M1000 (TECAN) set at 450 nm.

### SDS-PAGE and western blot analysis

EV protein content was determined by the bicinchoninic acid assay. Equal amount of proteins were resuspended in Laemmly sample buffer and separated via SDS-PAGE under non-reducing conditions for the detection of CD9, and CD63 or under reducing conditions for the detection of Alix and TSG101. Proteins were transferred onto a nitrocellulose membrane, incubated with 5% non-fat powdered milk in TBS-T (0.5% Tween-20) for 1 h and then with the following antibodies: anti-CD9 (1:1000, HansaBiomed Life Sciences), anti-CD63 (1:20,000, BD Pharmingen), anti-TSG101 (1:1000, Abcam, Ab83, 4A10), anti-GAPDH (1:1000, Santa Cruz Biotechnology). Secondary HRP conjugated antibodies (anti-mouse/rabbit IgG HRP-linked whole antibody donkey, GE Healthcare) were used and immunoreactive bands were visualized by using the Enhanced Chemiluminescence (ECL) (Merck Millipore). The signal intensity on the blot was further analyzed and quantified by densitometric analysis using ImageJ software (ImageJ bundled with 64-bit Java 1.8.0_172 downloaded from https://imagej.nih.gov/ij/).

### Real time RT PCR

EV-associated RNA was isolated from each purified EV pellet using an RNA Isolation Kit, according to the manufacturer’s protocol (HansaBiomed Life Sciences). The RNA concentration was too low to be reliably measured by fluorometric measurement (Qubit RNA HS assay, Thermo Fisher). To evaluate the expression of EV associated RNAs Real-Time PCR was performed in triplicates using miScript II RT Kit Qiagen for generation of cDNA, used subsequently as a template for PCR assays on SYBR Green technology employing QuantiTect Primer Assays (Qiagen) for β-actin and PSA mRNA. For ENY4 amplification following forward and reverse primer pair sequences (5′–3′) were used: GTCCGATGGTAGTGGGTTA and AAAGCCAGTAAATTTAGC. To assess miRNA expression, we used miScript primer assays (Qiagen) for individual miRNAs of interest or TaqMan® Array Human Micro- RNA A þ B Cards Set plus megaplex Pre-Amp primers (Thermo Fisher) for identification of miRNA signatures. All PCR reactions were run in CFX96TM Real-Time PCR Detection System (Bio-Rad, Hercules, CA, USA) using 96-well reaction microplates (Bio-Rad, Hercules, CA, USA), and analyzed by CFX Manager SoftwareTM to calculate threshold cycles (Ct). To appreciate variations in RNA expression results are expressed either (1) using a comparative Ct method. In particular, Ct values are converted into quantities in a linear scale (assuming amplification efficiency of 100% for each mRNA, (2(40−Ct)); or (2) using the ΔCt and/or ΔΔCt method, with RNA extracted from EVs isolated from Prostate Cancer LnCAP cell cultures used as a reference sample. These reference EV samples are currently available from HansaBiomed Life Sciences.

### Statistical analysis

Statistical analysis has been performed by using online available software for data analysis (www.icalcu.com). Briefly, we applied one-paired ANOVA test for group comparisons when appropriate, followed by followed by Tukey or Bonferoni post-hoc test. Details are provided in legends for each data figure.

### Ethics approval and consent to participate

The study was conducted according to the guidelines of the Declaration of Helsinki, and approved by the Institutional Ethics Committee of San Raffaele hospital (protocol code URINEBIOMAR, date of approval 04/08/2016). Written informed consent was obtained from all subjects involved in the study.

## Results

### High stability of EV content during storage and handling at room temperature

The major source of variability in the biomarker discovery and validation are pre-analytical decisions regarding sample harvesting, storage and processing. The current consensus protocols for urine EVs favor storage at − 80 °C although freezing and thawing can cause some EV loss or artefacts, such as EV aggregation or selective loss of larger EVs (MVs)^2^. In this study we have tested the use of a commercially available preservative that claims the conservation of urine RNA/microRNA/DNA and proteins for more than 2 years at room temperature (RT). We have focused on small nanosized vesicles (nsEVs) that are reported to be remarkably resistant to freeze–thaw cycles^19,20^. These are likely enriched with exosomes, while possibly containing a fraction of vesicles that do not originate from multivesicular bodies. The stability assessment was done on samples from 3 healthy donors, with 2 sample aliquots independently processed from each donor, overall featuring 6 biological replicates.

**Figure 1:**
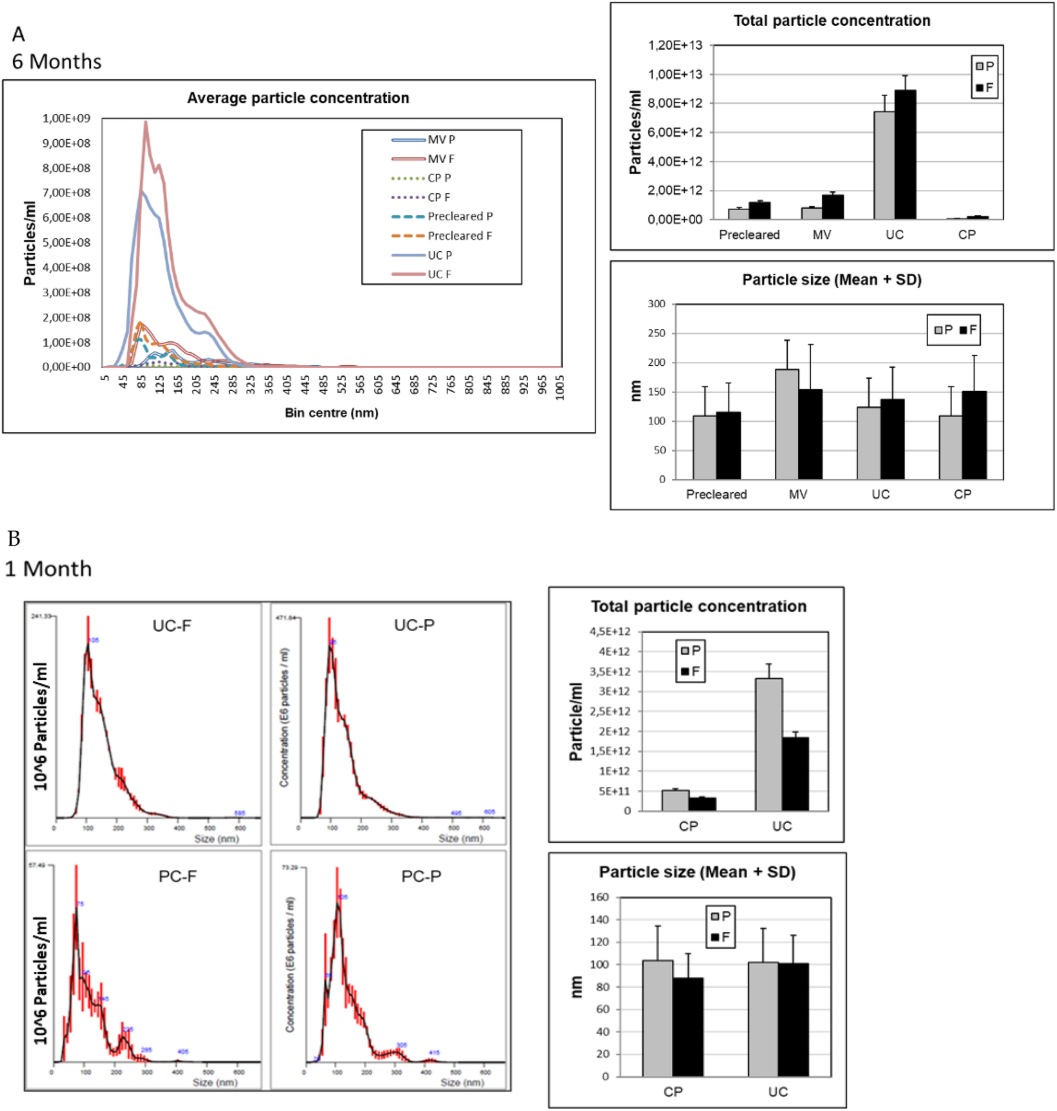
Counting and sizing of extracellular vesicles (EVs) isolated from urine preserved in different conditions. EVs were purified from urine obtained from three healthy volunteers by differential ultracentrifugation (UC) and chemical precipitation (CP) and was either preserved at − 80 °C (F) or adjuvated with the preservant at room temperature (P). Microvesicles (MV) were included in the analysis. Samples from three healthy donors, and 2 independently processed aliquots from each donor, are included in the assessment. Nanoparticle tracking analysis (NTA) analysis shows particle concentration and size as mean + SD after 6 months (**A**) and 1 month (**B**) of storage. The data analysis is done using a non-parametric test for multi-group comparison namely 1-WAY ANOVA followed by Tukey post-hoc test, no significant differences (p-values < 0.05) were observed.

**Figure 2:**
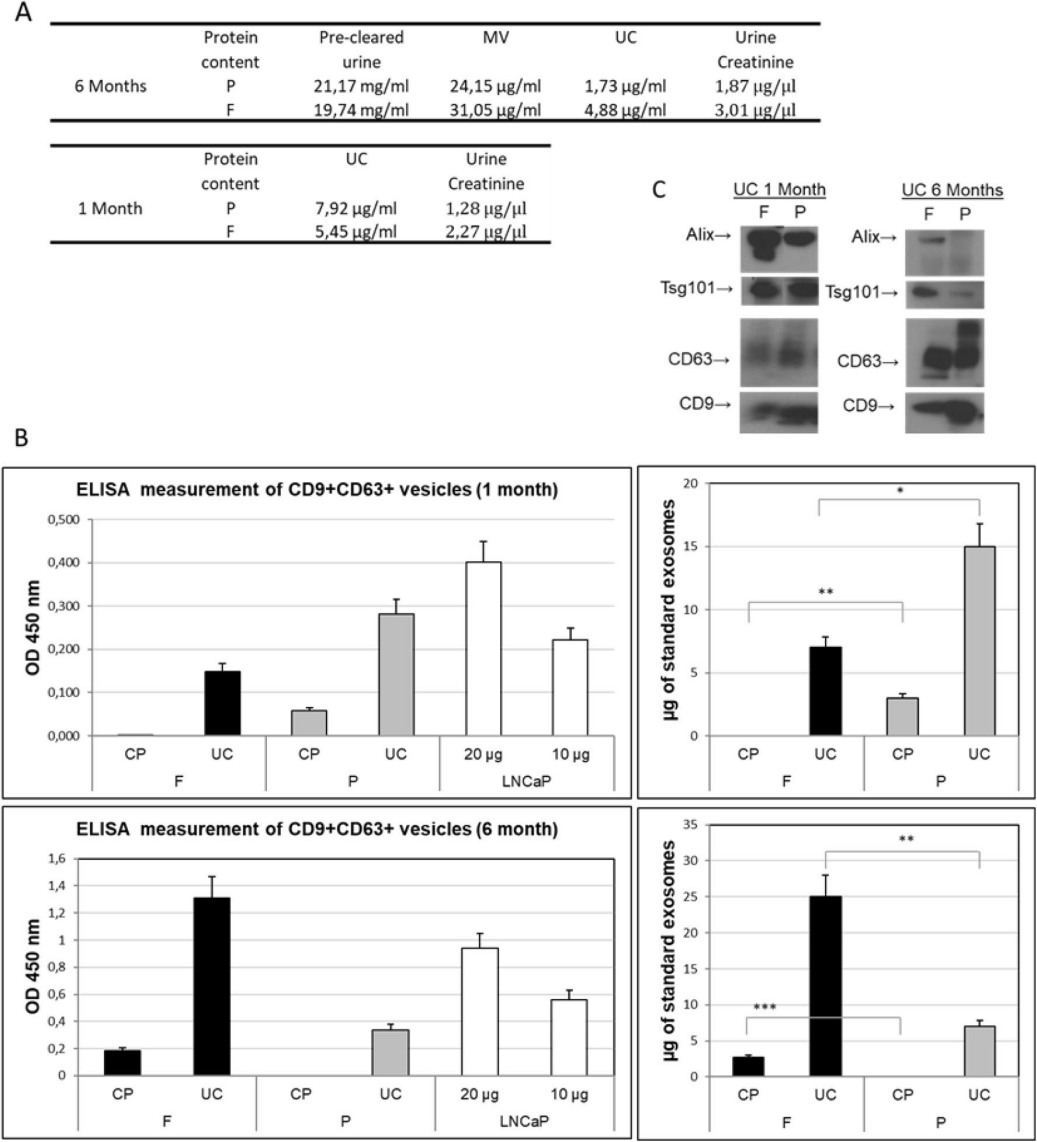
Quantitative analysis of EVs protein content. EVs were purified from urine obtained from three healthy volunteers, and 2 independently processed aliquots from each donor. The urines were either frozen at – 80 °C (F) or adjuvated with the preservant and stored at room temperature (P), After 1 and 6-months long sample storage, EV isolation was done by differential ultracentrifugation (UC) and chemical precipitation (CP). The Figure shows the total protein content measured by BCA assay, and creatinine content determined in whole urine and in urine microvesicles (MV) pellet and EV isolates from a single representative donor (**A**). For the same donor samples the specific content of canonical EV proteins was addressed by Western Blot (**C**, namely CD63, CD9, Tsg101 and Alix) (**C**) CD63 and CD9 expression was also measured for all analyzed samples by an ELISA assay (BEVs prepared from LNCaP cell conditioned medium were used as standards for relative comparisons in ELISA assay. A non-parametric test for multi-group comparison (three replicates each) 1-WAY ANOVA followed by Tukey post-hoc test was used for analysis of ELISA data; All p-values < 0.05 are marked with *, < 0.01 with **, < 0.001 with ***.

**Figure 3:**
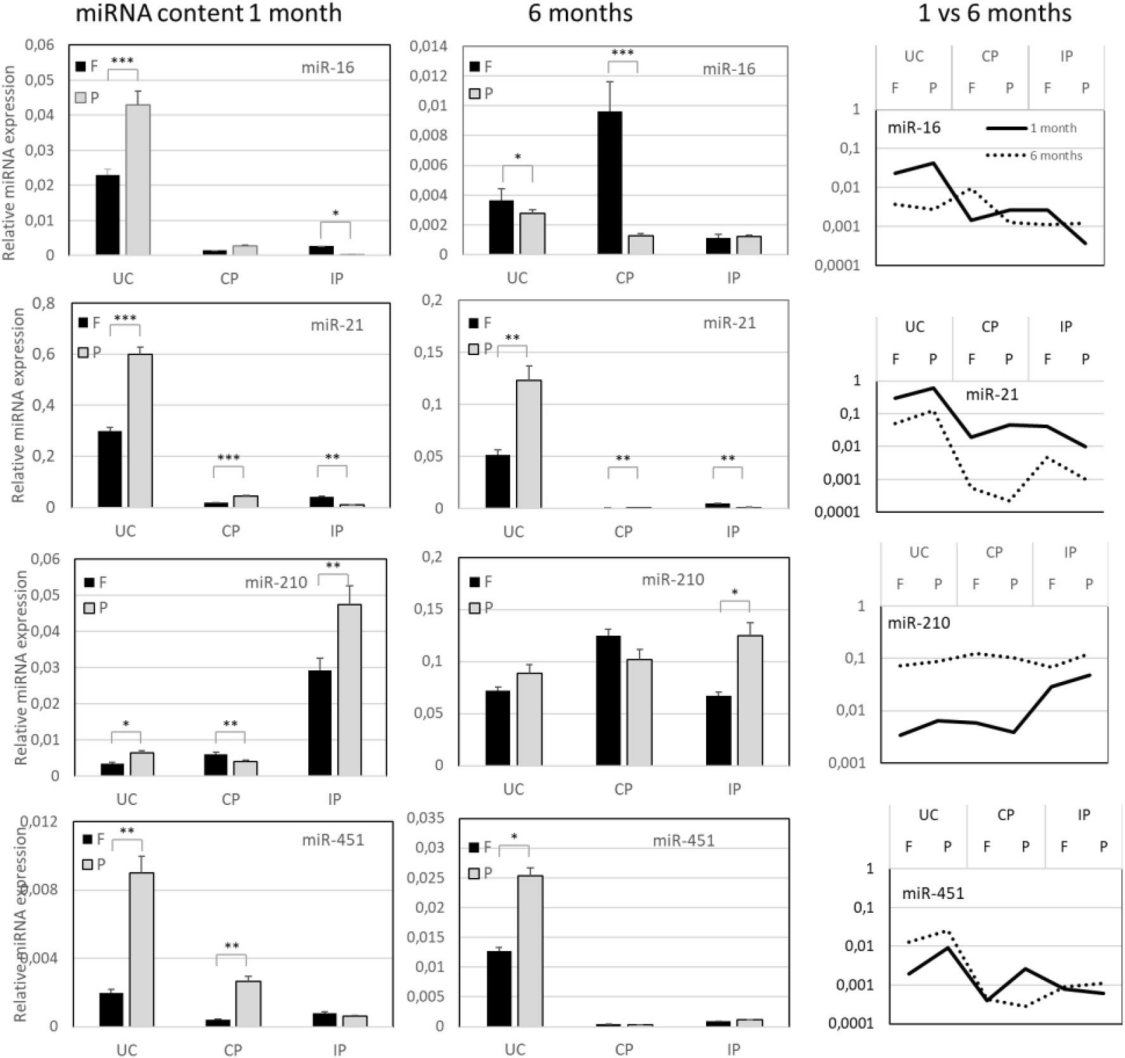
Quantification of EV miRNA content. Total RNA is extracted from EVs purified from healthy donor-obtained urine samples (N = 3), stored either preserved at room temperature (P) or frozen (F), using ultracentrifugation based protocol (UC), chemical precipitation (CP) or immuno-precipitation with anti-CD9 coated beads (IP). RT-qPCR amplification is performed of miRNAs de-scribed to be contained in EVs, namely miR-16, miR-21, miR-210 and miR.451. We show the average relative miRNA expression from three biological replicates. Relative expression was calculated with respect to a reference sample with known and constant RNA input (EV RNA from LnCAP cell culture). Samples from three healthy donors, and 2 independently processed aliquots from each donor, are included in the assessment. 1-way ANOVA and Bonferroni post-hoc test were used to determine statistical significance of observed differences * indicates p-value < 0.05, ** p-value < 0.01 and *** p-value < 0.001.

To compare the stability of exosome-like particles concentration and overall protein content we have measured particle number via NTA analysis in pre-cleared urine, and in reconstituted MV and exosome pellets from UC and CP samples. We have also processed the same samples with immunobeads based IP protocol (as explained in “Study design” in “Material and methods” section) but beads-bound EVs don’t allow for analytical methods such as NTA or protein content measurement. That is why in experiments represented on Figs. 1 and 2 only UC and CP were compared while all three methods were used for EV miRNA extraction and quantification (Fig. 3). As expected from our prior experience, UC gave major EV yields. We could also appreciate significant detection of exosome-sized particles (mean size around 100 nm) in reconstituted MV fractions confirming the prior observed co-pelleting of also nanosized EVs with larger vesicles at lower centrifugation speeds. In samples analyzed after 6 months, the overall EV concentration and size do not appear significantly affected by the storage although the number of retrieved vesicles is a bit lower upon exposure to room temperature (RT) (Fig. 1A). Conversely, in one-month-old samples the EV concentration is better maintained at RT with respect to frozen samples (Fig. 1B).

NTA measurements were quite in line with the determination of overall protein content (Fig. 2A). After 6 months UC pellet had higher values in frozen samples (2.8-fold increase) with the trend that was the same but more pronounced with respect to the same samples measured in NTA (1.3-fold). The overall urine protein content did not seem affected by different storage conditions while higher creatinine levels were measured in frozen with respect to RT samples (1.6-fold). After short term storage instead, protein content of exosome UC pellet was significantly higher in samples stored at RT (1.5-fold), while urine creatinine levels had an opposite drift and dropped 1.8-fold respect to frozen samples. The creatinine variations did not result significant, but the opposite trend observed for creatinine with respect to EV protein content raised questions about the suitability of creatinine as a calibrator of EV protein content in urine. This issue was therefore further addressed in the second part of this study. Observed data on EV proteins preservation overall supports the use of RT storage of urine samples especially for 1 month’s storage.

We have further assessed the amount of acknowledged EV and exosome markers by common quantitative assays such as ELISA and Western Blot. Sandwich ELISA assay measuring the level of small vesicles (co)expressing CD9 and CD63 showed correlation with overall protein content and vesicle number measurement (Fig. 2B and Supplementary Fig. 1). CD9 and CD63 are exosome enriched tetraspanins known to be well expressed on urinary vesicles^4,6,10,21^. The signals were higher (over three-fold) in samples conserved as frozen for 6 months, while, conversely, more prominent (~ two-fold) in those samples stored for 1 month at RT. Western blot analysis of UC pellet from the same samples showed concordant data with respect to the content of *bona fide* exosome proteins Alix and Tsg101 (Fig. 2C). The opposite trend for tetraspanins CD9 and CD63 revealed by WB in long term (6 months) conserved samples raises the possibility of contamination of pellets with membrane fragments and would be in line with compromised integrity of vesicles and surface displayed proteins upon long term storage at RT.

The EV RNA content is one of the fundamental indicators of vesicle integrity and functionality, being RNAs both putative biomarkers and effectors in pleiotropic functions exerted by vesicles in health and disease^3,7,9^. We have assessed the stability of overall RNA content of vesicles pelleted from differentially stored urine samples, with a particular focus on a most abundant RNA category found in EVs, small ncRNAs. We have attempted to use Qubit 2.0 Fluorimeter (Qubit Small RNA kit) in addition to Bio-analyzer concentration estimates in order to quantify the levels of total RNA in urine EV samples. Bioanalyzer RNA profiles were in line with > 80% abundance of small RNAs (miRNA) in all samples, but RNA content resulted too low to enable accurate Qubit 2.0 measurements, with some samples containing < 250 pg/μl. The latter was expected as our samples were obtained from urine volumes as low as 1 ml^11^. Therefore, we could not use a fixed RNA amount, but have decided to use an equal original urine volume for input normalization n amplification reactions. Such decision is in line with the common practice in diagnostic sampling^22^. MiRNAs are among the exosome cargo molecules that have elicited substantial interest and have been early approached for potential clinical interest. Different miRNAs have been described as associated to urinary vesicles in healthy or diseased subjects^6,12,23–25^. For the purpose of stability testing in this study we have first picked up several miRNAs commonly expressed in EVs, also in urine^23,25^. Within 1 month of storage, notoriously abundant miRNAs such as miR-21, 16 and 210 are successfully amplified (with Ct values ranging between 23 and 33) from exosome-sized vesicles purified from 1 ml of urine and showed high stability in samples preserved at RT (Fig. 3). The content of analyzed miRNAs was quantified as relative expression to that of a reference sample with known and constant RNA input (EV RNA from LnCAP cell culture)^13,14^. As expected, miR-451 resulted low abundant but was still revealed in all the samples tested. Increased stability of EV RNAs upon storage at RT was confirmed also after 6 months, in particular in UC samples. All three employed methods gave the material that can be reliably amplified and quantified, with the lowest miRNA expression obtained from IP samples. This result has been anticipated by an acknowledged fact that immunoaffinity selects for EV subpopulations, trading off the yield for specificity and purity. Interesting exception is observed for miRNA 210 that resulted highly enriched in IP samples (Fig. 3).

**Figure 4:**
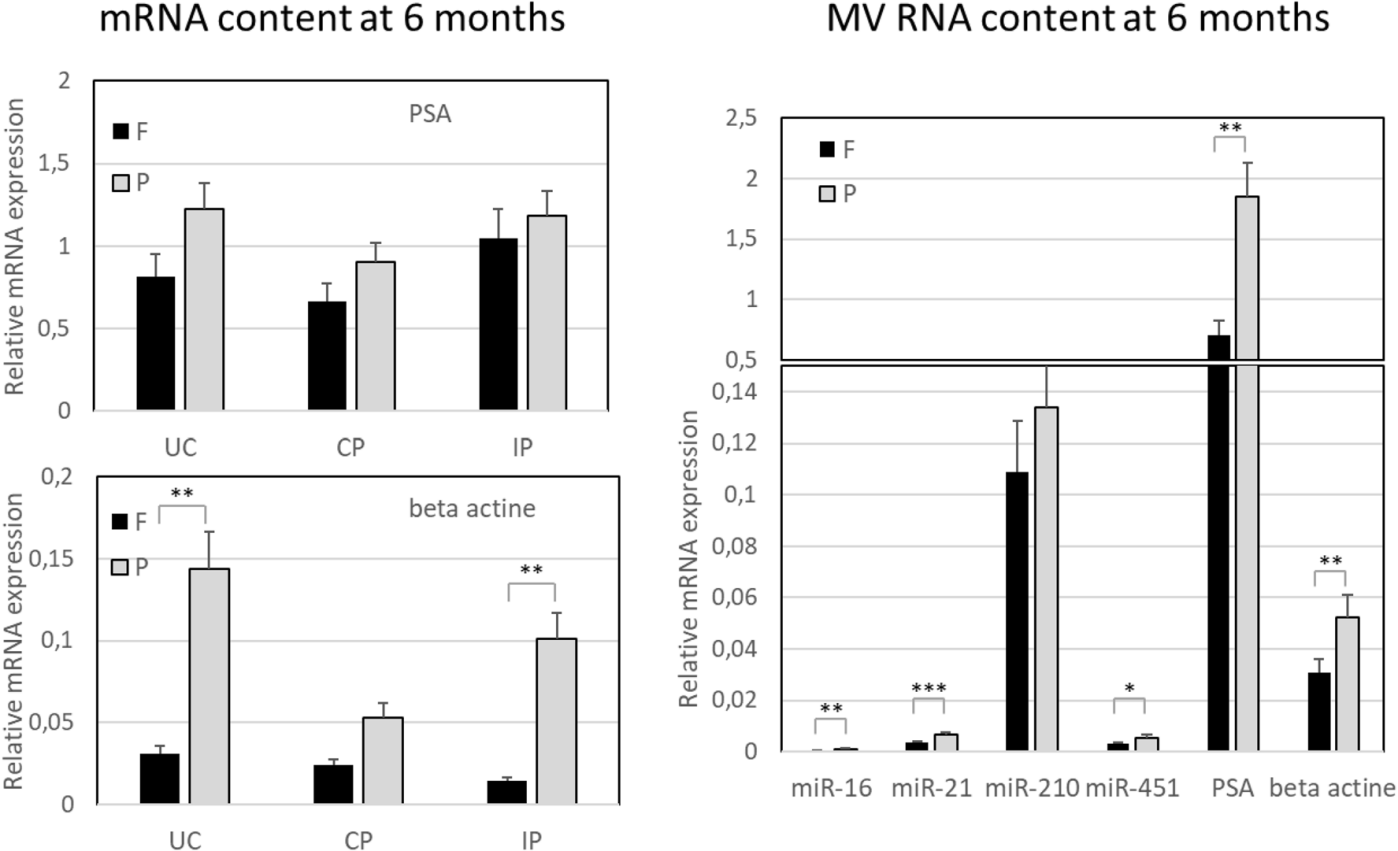
Quantification of EV RNA and MV RNA/miRNA content. Total RNA is extracted from MV pellets and from EVs purified from healthy donor-obtained urine samples (N = 3), stored either pre-served at room temperature (P) or frozen (F), using ultracentrifugation-based protocol (UC), chemical precipitation (CP) or immunoprecipitation with anti-CD9 coated beads (IP). RT-qPCR amplification of mRNAs prior described to be contained in urine EVs, namely β-actin and RNY RNA was done as de-scribed in Material and Methods. Both these mRNAs and miRNAs (miR-16, miR-21, miR-210 and miR.451) were analyzed also in MVs. Samples from three healthy donors, and 2 independently processed aliquots from each donor, are included in the assessment. One-way ANOVA and Bonferroni post-hoc test were used to determine statistical significance of observed differences * indicates p-value < 0.05, ** p-value < 0.01 and *** p-value < 0.001.

Certain incoherency in yield and stability between different RNA species was observed across different isolation protocols used. miR-21- and 16 levels were more in line with independently measured vesicle parameters (vesicle counting by NTA or protein quantification). Possible selective loss of some species and some vesicle subsets may be dependent on isolation protocol, as prior re-ported^3,15^. Noteworthy the advantage of RT preservation over freezing of urine was in particular evident for recovery of mRNAs after 6 months of storage (Fig. 4) as well as recovery of MV RNA content. In this study, we have assessed two RNAs widely reported in biofluid EVs (beta-actin) and in urinary EVs (PSA). The samples used for these experiments came from male donors, and PSA mRNA features as indicator of prostate derived vesicles. Overall, we can observe that preservation of urine at RT for sub-sequent EV RNA analysis is a good option after both long- and short-term storage. While long term storage at RT is not viable strategy for protein analysis, pursuing it in a short time setting (within a month) could be excellent solution favoring the stability, easy handling and shipment of urine samples for comprehensive EV analysis. Side indication from the first set of data that warrants more in-depth study is that both storage and isolation method could introduce a bias for loss or recovery of distinct EV populations in urine.

### Normalization strategies for quantitative analysis of urinary EVs

In the second part of the study we have addressed several normalization options that would enable the quantification and interpretation of differences in exosome/EV proteins and RNAs abundances in the context of intrinsic variability of concentration of urine, and urine EV. In order to make a best choice that fits our scope of proposing the method feasible both for research and clinical practice, we have considered the following premises. Though for quantitative comparisons a 24-h urine collection might be desirable, collecting first morning urine is definitely a more practical and conventional procedure that guarantees higher patient compliance. Moreover, studies on proteinuria have shown that this method of collecting random urine samples is valid and that the rate of protein excretion correlates well with that found in 24-h urine^16^. Other frequent pre-analytical choice in common diagnostics is that the samples are volume-normalized. In case of blood testing, due to a strict homeostatic control, testing and comparing the same volumes of samples is a standard option. In urine, this approach leads to inconsistencies in quantification of EV markers. We have first assessed exosome concentration and content of acknowledged vesicular markers in longitudinal samples with the aim to identify the reliable and measurable EV parameters that can be used for normalization. To this purpose we have used a small number of proband samples from 2 healthy donors, one male (Caucasian, 43 years) and one female (Caucasian, 45 years), from which the urine samples have been collected in 3 consecutive days. Each sample was divided into two aliquots that were independently processed, increasing thus the number of biological replicates and decreasing the impact of a random operational error to a small sample size.

**Figure 5:**
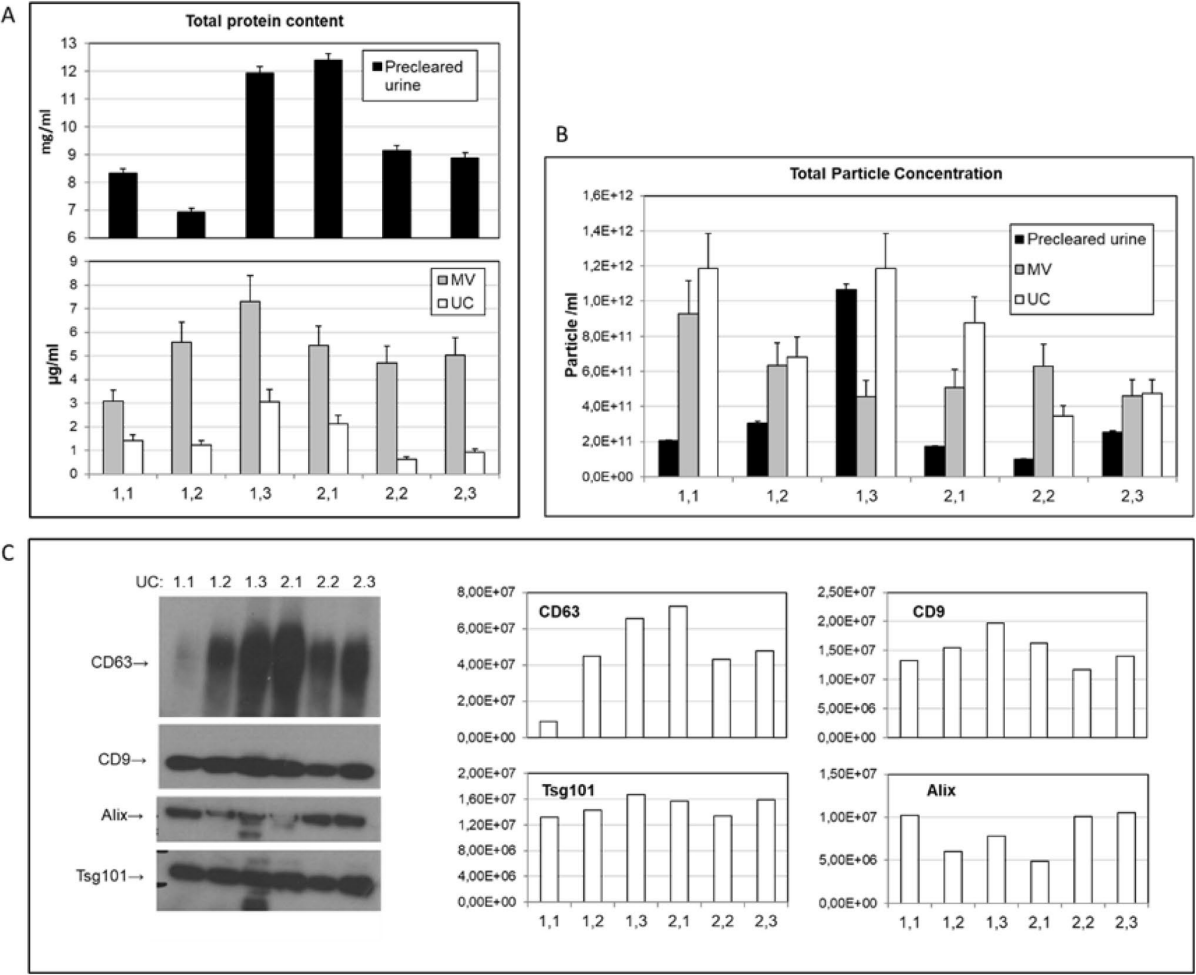
Comparative analysis of EVs content in longitudinal urine samples. EVs (UC) and MVs were purified by differential (ultra)centrifugation-based protocol from urine obtained from two healthy volunteers, one female (marked as 1) and one male (marked as 2), 45 and 43 years old, in 3 subsequent days. For each sample two aliquots of urine were processed independently and results presented as averages + SD. Total protein content was measured in a whole urine, MVs and EVs by BCA assay (**A**) particle concentration determined by NTA measurement (**B**) and EV proteins assessed by Western Blot (**C**). The signal intensity on the blot was further analyzed and quantified by densitometric analysis using ImageJ software and plotted on the right.

We have first measured exosome-sized particles concentration as well as protein content in both neat urine samples and in EV isolates prepared by ultracentrifugation. This isolation method was selected because it is still the major method used in urinary EV studies and has provided the best yield among the purification methods featured in this study. In addition, UC should not conceptually introduce a bias versus specific EV subpopulations, except for favoring the recovery of small EVs, while obtained EV isolates can be analyzed by different methods, including NTA, ELISA and Western Blot. NTA measurements revealed significant fluctuations of overall EV number within and between two healthy donors (Fig. 5A). Surprisingly, there was no correlation between EV counts in whole urine and in purified UC pellets while fine agreement was observed between protein content of whole urine and that of extracted exosome fractions (UC) (Fig. 5A, B and Supplementary Fig. 1). Analysis of canonical EV markers by Western Blot confirms day-to-day variations in EV protein content measured in the constant urine volume (Fig. 5C). Expression of *bona fide* exosomal markers Tsg101 and Alix apparently poorly correlated with a total protein content or vesicle counts and had different pattern from tetraspanins CD9 and CD63. To better assess individual protein patterns, we have performed densitometric analysis of gel bands (ImageJ, Fig. 5C) that highlighted CD9 signal as best reflecting other independent quantitative parameters of EV pellets (number and overall protein content of vesicles). Noteworthy CD9, according to densitometry, has excellent correlation with Tsg101 pattern but not with Alix pattern. Although CD9 is not exclusively confined to exosomes but is abundantly expressed also in MV (here not shown) it is known to be highly enriched in exosome-sized vesicles with respect to both parent cells and other vesicles subfamilies. The appeal of CD9 resides in the fact that it is displayed on the vesicle surface and thus can be exploited for isolation strategies and, of note for the focus of this study, for rapid vesicle quantification. As shown in Supplementary Fig. 1, the correlation between the EV protein content and the particle concentration measured in isolated small EVs is fair but not perfect (R = 0.781), while the EV protein content correlates better with the particle counts in the original neat urine (R = 0.817). In a similar way, also CD9 content was better aligned with the particle number measured in original neat urine (R = 0.860) than in EV pellets (R = 0.607), while it nicely correlated with protein content of isolated EVs (r = 0.931). Overall, among all independent indicators of EV content in urine, we found the best matching pattern between protein content of isolated EVs pellet, including CD9 measurement, and overall exosome-like (small EVs) particles counts in the original neat urine, Therefore, we retain these two to be most appealing EV-related parameters for mirroring their overall content.

Observed day-to-day variations in EV content are likely caused by diuretic effects. To equalize them, the samples can be normalized either i) using a volume correction factor based on a measurement of a parameter that reflects accurately EV-, or at least, urine concentration, without itself being additionally biased by any physiological of pathological variable, or by ii) using an optimal internal normalizer for each category of markers that are of our interest. Measurement of above identified EV related parameters can be used in both strategies. They can be used as preanalytical volume correction method prior to subsequent analysis of either proteins or RNAs in the EV preparations. They can also be used in second normalization option as the internal “housekeeping” EV markers. For objective protein biomarker measurement normalization to CD9 or Tsg101 level is a viable option, the choice of which would be based on methodological requirements (for instance an ELISA versus Mass Spectrometry, respectively). We will further discuss their use for normalizing the RNA expression data in the following chapter (Fig. 9).

**Figure 6:**
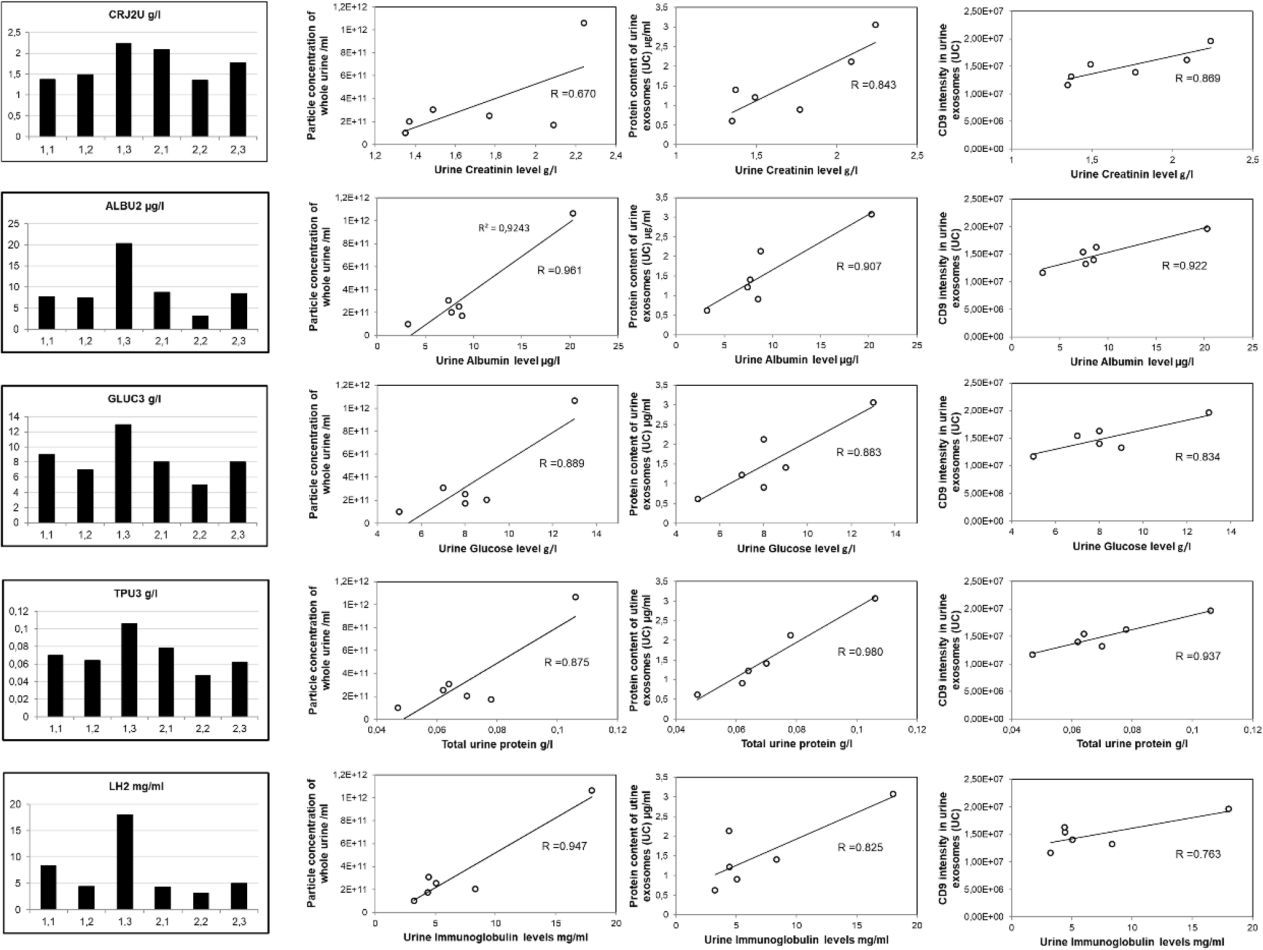
Analysis of urine biochemical parameters in longitudinal urine samples. Urine samples are obtained from urine obtained from two healthy volunteers, one female (marked as 1) and one male (marked as 2), 45 and 43 years old, in 3 subsequent days. These longitudinal urine samples were submitted to a routine biochemical urine analysis in a San Raffaele Hospital clinical laboratory within two hours from collection. Panels on the right show the correlation analysis performed by plotting the values of selected biochemical parameters against the EV parameters independently measured in the same urine samples. Explanation of acronyms used: CRJ2U, creatinine; ALBU2, albumin; GLUC3, glucose; TPU, total protein in urine; LH2, light immunoglobulin chains.

To explore the first option, besides EV-intrinsic factors, we have also considered some common biochemical parameters measured directly in urine as part of a standard laboratory analysis (Supplementary Fig. 2 and Fig. 6). We have included creatinine as it is frequently used for adjustment of urine analytes, including EVs, although its accuracy for the purpose has been long debated. To identify potentially useful biochemical indicator(s), we have first assessed the degree of association between each biochemical parameter measured across the longitudinal samples and the relative measurements of a particle number and a protein content of extracted urine EVs (UC) (Fig. 6).

Of all biochemical parameters measured in original urine samples the one that best correlated with the small EV content was the total protein content in urine (TPU), and the urine albumin level. TPU and Albumin correlated well with total particle counts in urine (R = 0.875/0.961), with urine exosome protein content (R = 0.980/0.907) and with CD9 content (R = 0.937/0.922) compared to creatinine correlation that gave coefficients of 0.670, 0.843 and 0.869 respectively. Other biochemical parameters which correlation to EV content is worth considering is urine glucose concentration and the heavy immunoglobulin chain levels (Supplementary Fig. 1).

**Figure 7:**
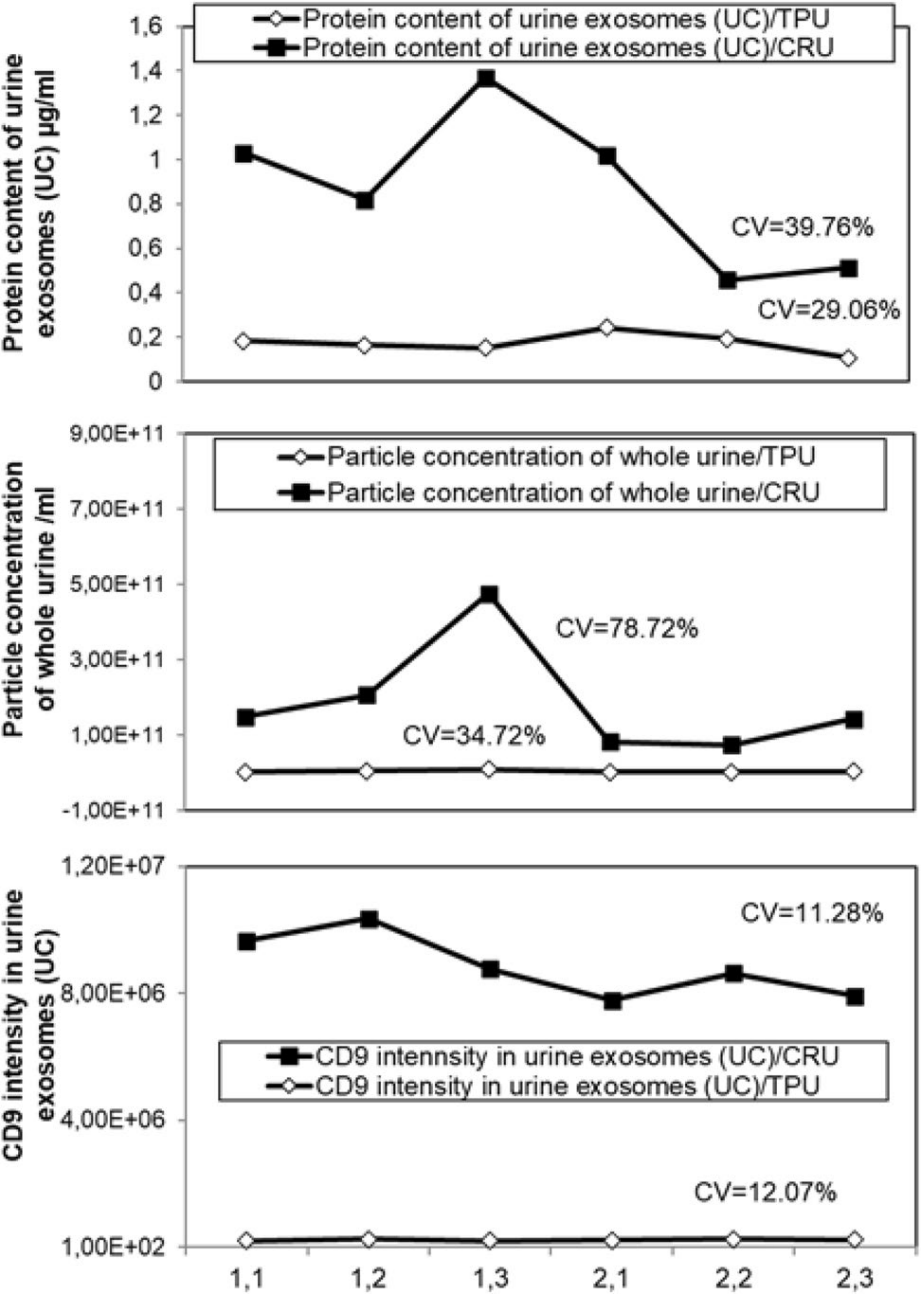
Normalization of EV content to urine biochemical parameters. Parameters retained most indicative pf urinary EV content in longitudinal urine samples, namely particle concentration in the whole urine, protein content and the CD9 signal measured in EV isolates, was normalized with urine biochemical parameters such as total urine protein (TPU) and creatinine (CRU). The scope of this normalization is to decrease the fluctuation of EV concentration, indicated here by the decrease of coefficient of variation (CV). Urine samples are obtained from two healthy volunteers, one female (marked as 1) and one male (marked as 2), 45 and 43 years old, in 3 subsequent days. The sample points are indicated in x axis.

Observed correlations opened to an interested possibility to use such routinely measured biochemical urine analytes to compensate for diuretic dilution effects and correct/normalize overall urine EV content (Fig. 7). We have considered the most promising those correction parameters that diminished most the variation between compared longitudinal urine samples. Coefficient of variation (CV) was calculated by dividing relative standard deviation and average of all samples. For instance, the correction with urinary TPU levels indeed reduced the variation coefficient between samples in terms of both urine particle numbers and urine exosomes protein content. Correction with urinary creatinine instead showed poor compensation effect.

### Internal normalizer-based strategies for urine EV RNAs

According to conventional approaches to elaboration and normalization of gene expression data, analysis of exosomal RNA content may benefit from some acknowledged internal normalizers comprising the RNA species that are found stably expressed across different human tissues and sample types and are reported to be sorted also into vesicles, though are not vesicle specific. The examples of latter include β-actin or GAPDH mRNAs, or some miRNA species including miR-16, miR-191 or miR-24^3,15,25,26^. In the past, small nucleolar RNAs (i.e. SNOU6) were often proposed for miRNA expression analysis, while the emerging policy is to normalize qPCR data using the same class of biomolecules.^27,28^ In order to cope with the complexity of the biofluids, it would be extremely beneficial to identify and use the RNA species that are enriched in vesicles of interest, in this case exosome-like vesicles. Moreover, depending on the purpose of the study, using RNAs that can normalize for organ specific vesicles could be considered. We have assessed the expression of 3 different mRNAs in the set of longitudinal samples, chosen as to represent different RNA categories relevant for possible normalization strategies (Fig. 7). Of note, mRNAs were present both in EV (UC pellets) and MV fractions of all samples (Fig. 7), differently from a protein content, that was too low and thus not detectable in MV pellets (not shown). Beta-actin was selected as a commonly used gene expression normalizer. PSA transcript was suggested as a potential prostate-specific mRNA that can have both the value of a normalizer or as a disease biomarker^29,30^. PSA mRNA has often been reported and assessed in urinary EVs. Finally, we have employed RNY4, a member of HY RNA family that have been detected as abundant RNA species in biofluids and in EVs^29,31,32^.

**Figure 8:**
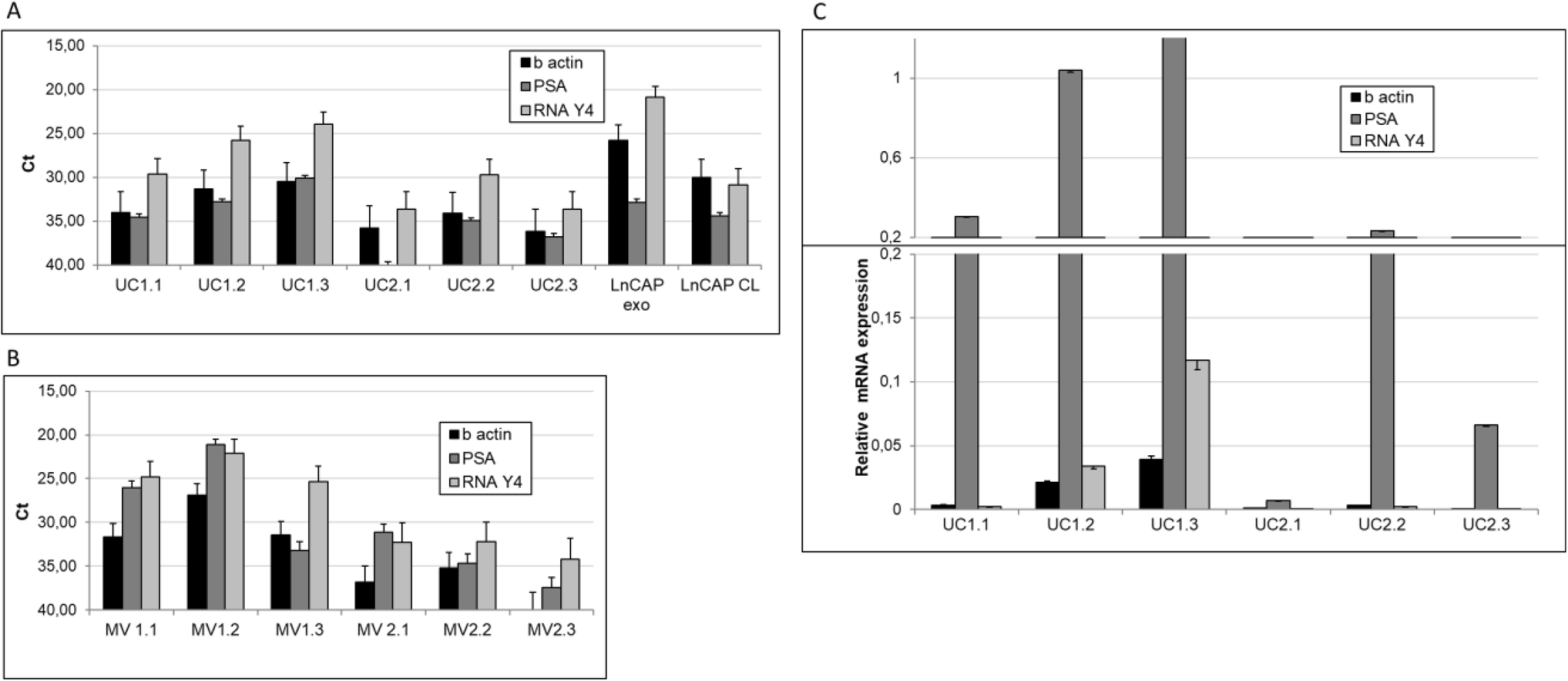
Detection of urine EV-mRNAs by RT-qPCR in longitudinal samples. Following ultracentrifugation, total RNA has been extracted from EV (UC) and MV pellets and RT-qPCR amplification of mRNAs of interest performed as described. Purification is performed on urine obtained from two healthy volunteers, one female (marked as 1) and one male (marked as 2), 45 and 43 years old, in 3 subsequent days. For each sample two aliquots of urine were processed independently and results presented as averages + SD. The results are shown both as Ct values (on the left) and as relative RNA expression (on the right) calculated as delta Ct versus reference sample (RNA extracted from Prostate Cancer LnCAP cell line exosomes).

All these mRNAs were detected upon RNA extraction from EVs, in a material corresponding to 1 ml of starting urine sample, with roughly similar trends observed across the experimental points (Fig. 8). Beta-actin, and, especially PSA mRNA, resulted not-abundantly expressed in analyzed samples. Poor expression of PSA mRNA would be consistent with its specific prostate origin and with the fact that we did not use any isolation method enabling specific enrichment of prostate derived vesicles. Surprisingly however, EV expression of PSA mRNA did not correlate with the gender of the donor. Higher signal for PSA mRNA was obtained in a female donor sample (1.1–1.3) raising thus doubts on the utility of this mRNA as a truly prostate specific marker. Instead, RNY4 was well amplified from all UC pellets. We have previously confirmed that this small RNA is multifold enriched in exosome-sized vesicles with respect to parent cells (~ ΔCt = 10). These two observations constitute premises to consider it a promising internal reference for urinary EV RNA analysis.

**Figure 9:**
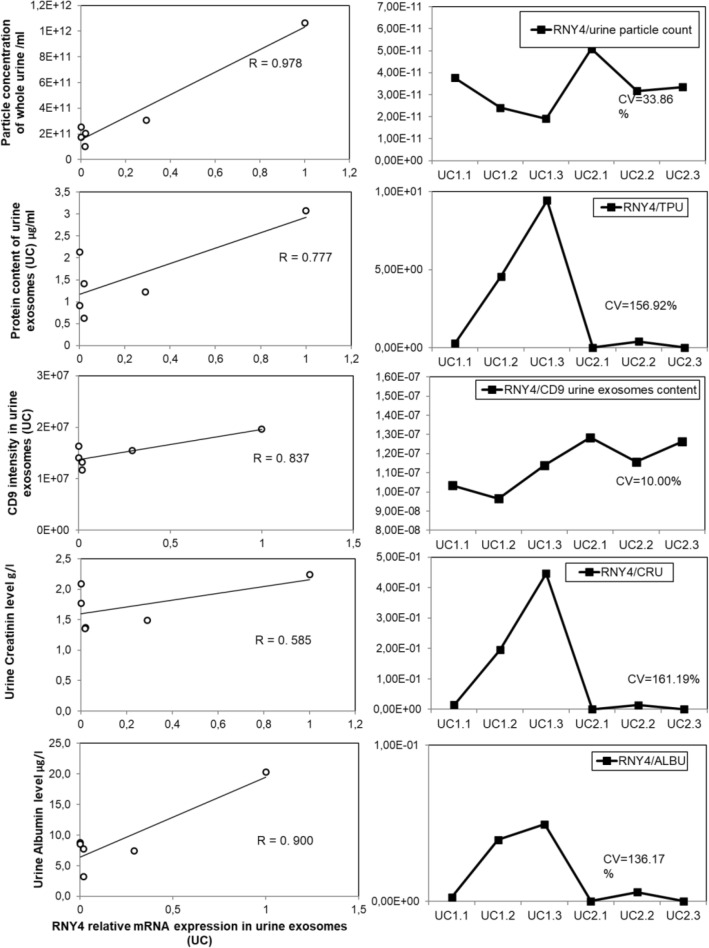
Normalization of EV-RNA content variations to urine biochemical parameters and EV number and protein content. RV RNY4 RNA expression levels across the longitudinal urine samples were first plotted against selected urine biochemical parameters, namely urinary creatinine, albumin and total protein content, as well as against independent EV content indicators, such as particle number measured in the whole urine and the CD9 signal measured in the respective EV isolates (on the left). The same parameters were subsequently used in the attempt to normalize/compensate for variations in EV RNA signals across the samples, revealed as decrease in coefficient of variation (CV, on the right). EVs (UC) were purified by differential (ultra)centrifugation-based protocol from urine obtained from two healthy volunteers, one female (marked as 1) and one male (marked as 2), 45 and 43 years old, in 3 subsequent days. The experimental points are indicated at the x axis of the right panel. The indicators shown on the relative y axis are matched between left and right panel.

At glance, absolute mRNA expression pattern (row Ct values measured) did not mirror the EV number and protein concentration measured in the same EV pellets (comparing Fig. 5 and Fig. 8). On the other hand, transforming Ct values into quantities (using comparative Ct method) (Fig. 8C) or converting them into a linear scale (assuming amplification efficiency of 100% for each mRNA, (2^(40−Ct)^)) reveals the pattern that correlates perfectly with overall small EV content in the urine (as observed in Fig. 5). RNY4 was most abundantly expressed in all samples and correlated with overall particle counts in urine, and with protein and CD9 content in urinary EVs with respective coefficients of correlation of R = 0.987, R = 0.0777 and R = 0.837 (Fig. 8). Noteworthy, relative RNY4 expression correlated well with urine total protein, in particular albumin content (R = 0.900), rather than with creatinine levels (R = 0.585) (Fig. 9). When we attempted to correct the RNY4 expression inter-sample variations by normalizing it with creatinine or urine total proteins^33^, we did not observe the decrease in inter-sample CV, while the best correction is obtained by considering the respective CD9 levels in the sample. The latter decreased overall inter-sample variation to less than 10% (Fig. 9). This supports the use of CD9 as a “housekeeping” EV marker to correct for urine dilution-related variations in EV-RNA content.

**Figure 10:**
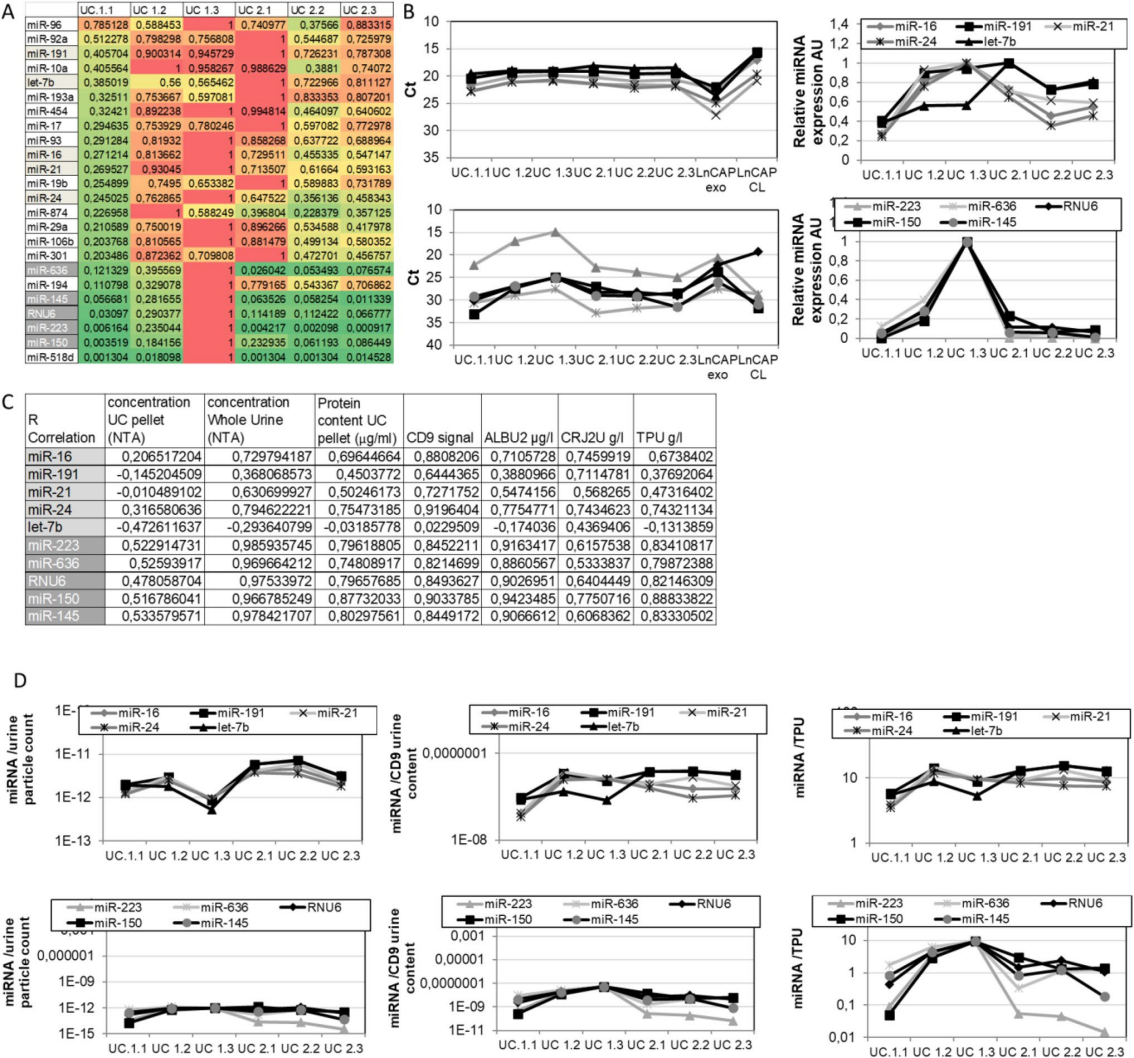
Detection urine of EV-miRNAs by RT-qPCR in longitudinal urine samples. Following ultracentrifugation, total RNA has been extracted from EV and MV pellets and RT-qPCR amplification and quantification of a 24 miRNA Taqman Custom Array performed as described. The results are expressed both as Ct values (**B**) and as relative RNA expression (Table **A**, and panel **B** on the right) calculated as delta Ct versus reference sample (RNA extracted from Prostate Cancer LnCAP cell line exosomes). The correlation index was determined between the trend observed for miRNA expression levels across the samples and the respective values for selected EV parameters (particle concentration and protein content) as well as biochemical parameters measured in the same urine samples (creatinine, albumin and total protein content) (**C**). Finally, the normalization of miRNA expression levels at longitudinal points is attempted using best correlating EV and urine parameters (**D**). EVs (UC) were purified by differential (ultra)centrifugation-based protocol from urine obtained from two healthy volunteers, one female (marked as 1) and one male (marked as 2), 45 and 43 years old, in 3 subsequent days. Experimental points are shown on relative x axes. For each sample two aliquots of urine were processed independently and averaged.

In the last part of our study, we tried to challenge our approach for identification of best EV normalizers by applying it to a commercial custom miRNA array. We have chosen the array featuring 24 miRNAs, including both putative normalizers for diverse tissues and biofluids, as well as putative markers for diverse diseases, including cancer, that we have already deployed for analysis of EV-miRNA expression in cancer plasma samples (not published). We have assessed this panel miRNA expression pattern in our small longitudinal samples set and we have identified two clusters (Fig. 10). The first one (cluster 1) comprises miRNAs that are notoriously and abundantly expressed across different human samples and tissues (i.e. miR-16, -21, -191, -24). By evaluating Ct values for the same urine volume input, these seem to be uniformly expressed in all samples regardless the prior observed variations in EV concentrations. In a control experiment with LnCAP culture-derived EVs, we noticed that these miRNAs were not enriched in EVs with respect to a respective parent cell lysate (Fig. 10B). Conversely, the levels of a second group of miRNAs encompassing miR-223, -150, -145. -636 and RNU6 (cluster 2) changed across the longitudinal samples and appear enriched in control standard EVs with respect to respective cells. Relative miRNA expression levels correlated better with the inter-sample fluctuations observed in EV number and protein content, in particular for miRNAs belonging to the second cluster (Fig. 10A and C). Best correlation of EV-miRNA expression was noticed with respect to overall particle counts in original urine (R = 0.967–0.986 for mRNAs encompassing miR-150, -636, -145 and -223, compared to R = 0.368–0.795 for miRNAs such as miR-191, -21. -21, -16) and with respect to CD9 levels in extracted urinary EVs (R = 0.821–0.904 for first cluster and R = 0.644–0.920 for the second one) (Fig. 10C). Accordingly, the relative expression of the miR-150, -636, -145 and -223 miRNA cluster (cluster 2) was well associated to urine albumin level (R = 0.886–0.943) but not to urine creatinine (R = 0.533–0.775), unlike the cluster comprising miR-191, -21. -21, -16 expression (cluster 1) that showed only week correlation to urine biochemical parameters (R = 0.381–0.775 for albumin and R = 0.568–0.746 for creatinine) (Fig. 10C).

We ultimately attempted to compensate the inter-sample variations of miRNA expression in EV preparations by normalizing the results by EV parameters, with the best results obtained when the particle count measured in the neat urine and the CD9 content in urine were used (Fig. 10D). Such a result was coherent with our prior described results.

Overall, such an approach would point out the cluster 2 as an appealing miRNA panel for further validation as urine EV miRNA content calibrator in the extended sample cohort.

We wanted to compare our approach and results to a widely used and acknowledged method for identification of reliable internal controls for normalization of miRNA/gene expression data. This method consists in the use of statistical algorithms that rank the reference genes by calculating their overall expression stability, in associations with other possible control genes. We indeed applied three of the most used and often combined software solutions, namely GeNorm, NormFinder and BestKeeper (supplementary Fig. 3A–C) to the input data comprising either relative expression (quantities) or absolute expression (Ct values) data collected from our pilot sample set, according to each algorithm requirements^34^. The key assumption of all these algorithms is that the investigated candidate reference genes do not vary in their expression systematically across the samples being considered. Even if this is true in terms of impact of different physiological or pathological conditions, in the scenario of identification and implementation of urinary vesicles biomarkers, this assumption is violated in practice by day-to-day fluctuations in diuretic dilution of the urine. If we pursue the scenario applied in this study, featuring the use of the same input volumes of the samples, without normalizing the input of cDNA for RT-PCR, then the use of computational algorithms is innately flawed. The measurement of RNA/cDNA extracted from EVs isolated from low original urine volumes (1 ml) is difficult and imprecise even with the use of Qubit assays, and is time consuming and costly for routine or high throughput analysis. Indeed, all three algorithms employed to analyze miRNA expression data in a pilot set of longitudinal samples (6 experimental points, 12 independently processed samples), would indicate as most stable genes miR-24 and miR-16 that are apparently stable across the all samples without being impacted by the real differences in EV concentration. This approach has clear limitation for selection of reliable internal references in this case, where the lack of statistical effect does not account for the sample specific variation and is thus missing the specific problem addressed by this study.

## Discussion

The present study addresses some fundamental, yet not resolved issues in the analysis of urinary EVs and there-associated content, in particular RNAs, regarding the bias due to preanalytical variables and inappropriate normalization strategies. While resolving these nods would bring benefit to the research, biomarker discovery and diagnostics development, we tend to privilege the solutions that are likely to offer major compliance with current clinical lab practices in terms of timing and processing of urine samples. At the same time, the protocols that would facilitate the sample handling for storage and transport, as to serve the needs of biobanking and multicentric studies, are also addressed. To this prospect in this study we focused on “random” urine samples rather than 24 h urine collection, as the former are common practice in clinical consultations and biobanking, and are proposed to correlate well with 24 h urine for their protein excretion rates^16^.

In EV research, preanalytical variations are exacerbated by the fact that the field is relatively new and there is still an attachment to “lab specific” protocols and overall lack of consensus. This impacts in particular the multicentric collaborative studies and the use of biobanked samples for EV markers validation. While intrinsic and extrinsic biological variations between samples (i.e. age, gender, circadian rhythm, body mass index, medication, current sickness etc.) should be carefully considered when data are interpreted, technical variations introduced during the collection or processing need to be controlled and hopefully excluded. The stability of once purified small EVs, including exosomes, stored as frozen at prolonged times is already well documented^35^. However, storage of urine after the initial collection and prior to exosome/EV isolation and analysis may still be a challenging issue in the routine clinical analysis settings, and even more in the case that shipping of samples is required in the frame of large biomarker discovery efforts. To inhibit degradation and preserve stability of otherwise unstable urine analytes, usual clinical practice foresees that all urine specimens, unless analyzed within 2 h, are refrigerated during the collection and are kept frozen until the necessary analysis. Moreover, different preservatives and stabilizers are seldom applied including acid, base, formaldehyde, toluene, biocides or protein inhibitors^36^. Many studies along with our observations demonstrated that the urine storage at − 20 °C, that corresponds to standard short-term clinical sample storage, appears least favorable for EV recovery both due to a high degradation rate and the pronounced calcium phosphate precipitate formed at this specific temperature^2^. Indeed, the common consensus is that storage at − 70 to − 80 °C should be optimal. The fact is that the storage of large volumes and shipping of frozen samples for EV analysis can be costly and unreliable, possibly altering their stability. Freezing of samples can potentially cause loss of EVs either due to an enhanced aggregation of vesicles or their adsorption to the surface of the collection vessel. The further advantage of avoiding freezing of urine likewise other biofluids is the preservation of larger microvesicles that are typically lost during freeze-and-thaw cycles.

We report stability of urinary EVs and their content upon preservation at RT after 6 months as well as recovery and analysis of EV markers from 1 ml of original urine volume. The choice of a specific EV isolation technique is dependent on the type of downstream uses. We employed three single-step EV pull-down-based protocols, that all recovered quantifiable EVs and associated markers. We observed differences in yield and in relative ratios of different measured EV parameters, consistently with what reported in other studies. When detecting already associated-to-EV markers in low volume patient samples, it is possible to prioritize yield (like CP) or selected enrichment of EVs of interest (IA), as well as choose simplicity and low time-consuming solutions (offered by both). We take into consideration that a selective loss of some particular markers, such as some miRNA species, may occur dependent on different isolation protocols, and due to their different impact on different vesicle subpopulations in urine^3,15^. Preferential sorting of distinct miRNAs to specific vesicle populations, distinct by size or immunophenotype, has been reported^26^. In these pilot assessments we used ubiquitously expressed RNAs that are not confined to vesicles, though the evidence supports more robust overall miRNA profiling from urine vesicular fraction rather than directly from the whole urine^25^. This means that, although EVs are likely to contain the majority of urinary RNAs, analysis of hereby used preparations such as UC or CP pellets that contain also co-precipitated contaminants do not reflect only intra-EV content. To this respect, some studies suggest that the extravesicular RNA in urine is not recovered along with precipitated EVs, consistent with the high presence of ribonucleases in urine after UC^37^.

In this study we proceeded with UC as it balances a no-bias versus specific EV subpopulation, with a wide adoption of this EV isolation method for the quantification of a variety of common EV indicators. Finally, we addressed the suitability of different normalization strategies applied in preanalytical or analytical phase of the quantitative analysis of EV mRNAs and miRNAs. Such normalization is challenging both due to a huge inter- and intra-donor urine diuretic variation at longitudinal harvesting points, as well as EV intrinsic heterogeneity. Preanalytical and in-analytical normalization decisions may also lead to a generation of biased data and mask the recognition of rare and relevant EV biomarkers. It is clear that the requirements for expression analysis in urine EV may not match respective recommendations for tissue and cell derived RNAs, and may even differ from those for other biofluids EVs.

To our opinion preanalytical normalization based on the use of one of routinely measured urine biochemical parameters would be appealing in order to cope with urine concentration variations, and would enable due adjustment of either sample volumes or of measured quantities. The latter mode would be compliant with the way clinical diagnostics is done today which is volumetric: certain volumes of a biofluid samples are typically measured and the results compared to reference values. To-date many studies have proposed or questioned the use of creatinine for such purposes. Indeed, creatinine levels are not merely attributable to simple variations in urine dilution but rather to a kidney function, and can thus introduce a bias in inter-population or even longitudinal comparisons. This could be particularly evident in pathological conditions and might be missed by comparisons of healthy individuals. We have, instead, proposed other parameters, such as urine total protein content or albumin, that, unlike creatinine, correlated nicely with the independent indicators of urine EV concentration, namely particle number or EV protein content. Salts or glucose content also warrant further consideration, along with likely interrelated urine conductivity and gravity, that are besides creatinine, most frequently used proxies of urine concentration. However, also urinary proteinuria or albumin levels could be influenced by a particular pathological condition. We have to keep in mind that neither NTA measured exosome-like particles, nor protein content of UC pellet are purely attributable to EVs due to artifacts end co-pelleted contaminants, respectively. Such correction maneuver should be tested on an enlarged samples cohort, and possibly coupled to condition-specific EV biomarkers. We have done an exploratory study in which we probed large number of parameters in a small set of samples from healthy donors, and have detected consistent intra and inter-sample variations that led us to sort out those that are most promising for EV concentration normalization. In such a way this approach, while having obvious appeal and match with well-established standard clinical practice, will be feasible for validation in enlarged and specific sample cohorts, implementing optimized SOPs for urine sample preservations.

Use of internal controls, so called “housekeeping” genes, is the most acknowledged strategy for analytical normalization of gene expression analysis. However, we demonstrate that the application of strategies that are developed and commonly used for tissue samples, can be misleading for the identification of the right normalizer panels for EV RNA analysis in complex biofluids. This is in particular true for urine that, differently from blood, is “not buffered” for volume, concentration and pH variations. Therefore, we considered the best EV miRNA normalizers those miRNAs which longitudinally-assessed trend nicely mimicked independently measured EV concentration changes in the same samples. We have identified a potential normalizer panel that comprises differently abundant miRNAs, therefore suitable for different targets of interest. We do not consider this panel a universal solution but, rather, propose such an approach to evaluate and define the normalizer panels that are tailored to a specific research or clinical question.

There is some evidence supporting the low abundance and low occupancy model of sorting of miRNAs into small EVs^38^. This means that only a subset of the cell microRNAs gets packaged into exosome-like EVs, and the content, in terms of copies per vesicle, is reported to be low (much less than 1) even for abundant miRNAs such as miR 223. On the other hand, the sorting of certain miRNAs into EVs has been demonstrated to be specific^39^, either dependent on the EV source, or limited to some EV subpopulations^26^. For instance, miRNAs can co-sort with specific tetraspanins. This would nicely fit with the observed strong correlation with miRNA content and CD9 expression in urinary EVs, and can provide some implications for the development of EV isolation strategies aimed at specific enrichment of particular EV subpopulations and particular EV biomarkers.

Our data indicates that RNY4 is a good candidate as a part of an internal reference panel for the analysis of urinary EV RNAs. This RNA is transcribed by RNA polymerase III while protein coding genes are transcribed by RNA polymerase II. However, the RNA Pol III transcriptome display significant sequence complementarity to protein-coding genes and is suggested to interplay and regulate their metabolism and expression^40^. This small RNA might be adequate control for assessment of mRNA expression and warrant further confirmation studies. One aspect that has to be empirically determined for each candidate normalizer is the eventual pathological variation. For instance, HY RNAs are reported to be multifold up-regulated in some cancers or inflammatory diseases^41^. Evidence also suggests that part of the HY RNAs processing is occurring in the same EVs that are transporting them^42^.

It is noteworthy that EV content of CD9 is a good option for immunometric measurements that can be easily integrated with analysis of other EV markers (not only proteins) in urine, while Tsg101 remains an appealing candidate for sample normalization in the phase of biomarker discovery in mass spectrometry and metabolomic studies. The stability of expression of these exosomal markers across the samples could be challenged in specific pathological conditions such as cancer and inflammatory states, and should thus be carefully considered within specific biomarker studies. Also, the co-sorting of any particular marker of interest to CD9- or TSG101-positive vesicles should be ascertained, due to a heterogeneity in EV biogenesis paths and immunophenotypes.

## Conclusions

Body fluids are a never-ending source of biomarkers useful for diagnosis and prognosis of plenty diseases, including cancer. Urine in particular, due to the ease of collection, less complex content with respect to the blood and mostly reflecting the contribution of the urinary tract, guarantees patient compliance and longitudinal disease monitoring. On the other hand, the diuretic fluctuations and the numerous factors that can affect its volume and content, make difficult the interpretation of urine analysis results. For this reason, the preanalytical and postanalytical decisions regarding the processing and normalization of urine content are a key step in the biomarker identification and translation into a clinical practice. Since EVs have been considered a surrogate of cells that produce them and real-time sentinels of tissue homeostasis, the full realization of their biomarker potential critically relies on a correct understanding and a solution of such issues.

Overall, our study prompts both preanalytical and analytical strategies that are most compliant with established clinical procedures, without including extensive sample handling, and laborious EV purification or counting prior to analysis. This exploratory study design (independent sample processing for biological replicates, and assessment of large number of parameters in a small proband sample set) allowed for a pilot evaluation of longitudinal variations of biochemical and EV parameters in urine, providing candidates for specific urine EV biomarkers validation studies (small number of parameters in large sample cohorts).

## Supplementary Information

Supplementary Figures.

## Data Availability

All data supporting the conclusions of this research article are included within the manuscript.

## Abbreviations

EVs: Extracellular vesicles
NTA: Nanoparticle tracking analysis
MV: Microvesicles
RT: Room temperature
UC: Ultracentrifugation
IP: Immunoaffinity-based pull-down
CP: Chemical precipitation
