# Supplementary material for: Urine stabilization and normalization strategies favor unbiased analysis of urinary EV content
Source: Sci Rep. 2022 Oct 21;12:17663. doi: 10.1038/s41598-022-22577-3 (PMC9587215; doi:10.1038/s41598-022-22577-3)
Supplement: Supplementary file 1 — Supplementary Figures. [file 41598_2022_22577_MOESM1_ESM.pdf]

Supporting material

Urine stabilization and normalization strategies favor unbiased analysis of urinary EV content

Riccardo Vago[1,2], Giorgia Radano[3], Davide Zocco[3], Natasa Zarovni[3,4]

[1] Urological Research Institute, Division of Experimental Oncology, IRCCS San Raffaele Scientific Institute, Milano, Italy.

[2] Università Vita-Salute San Raffaele, 20132 Milano, Italy

[3] Exosomics S.p.A, 53100 Siena, Italy

[4] HansaBiomed Life Sciences OU,

This pdf contains:

- **Supplemental figures with their legends (Figure S1-S4)**

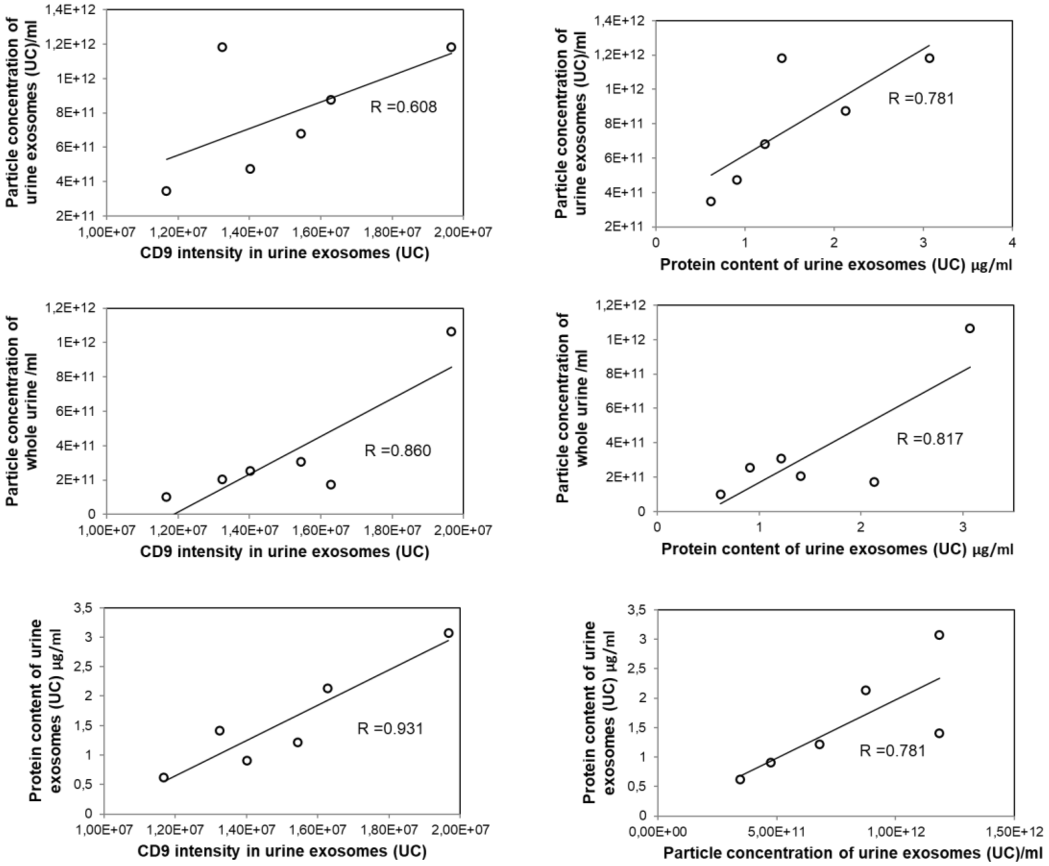

Supplementary Figure 1. Correlation between independently measured urine EV content indicators.

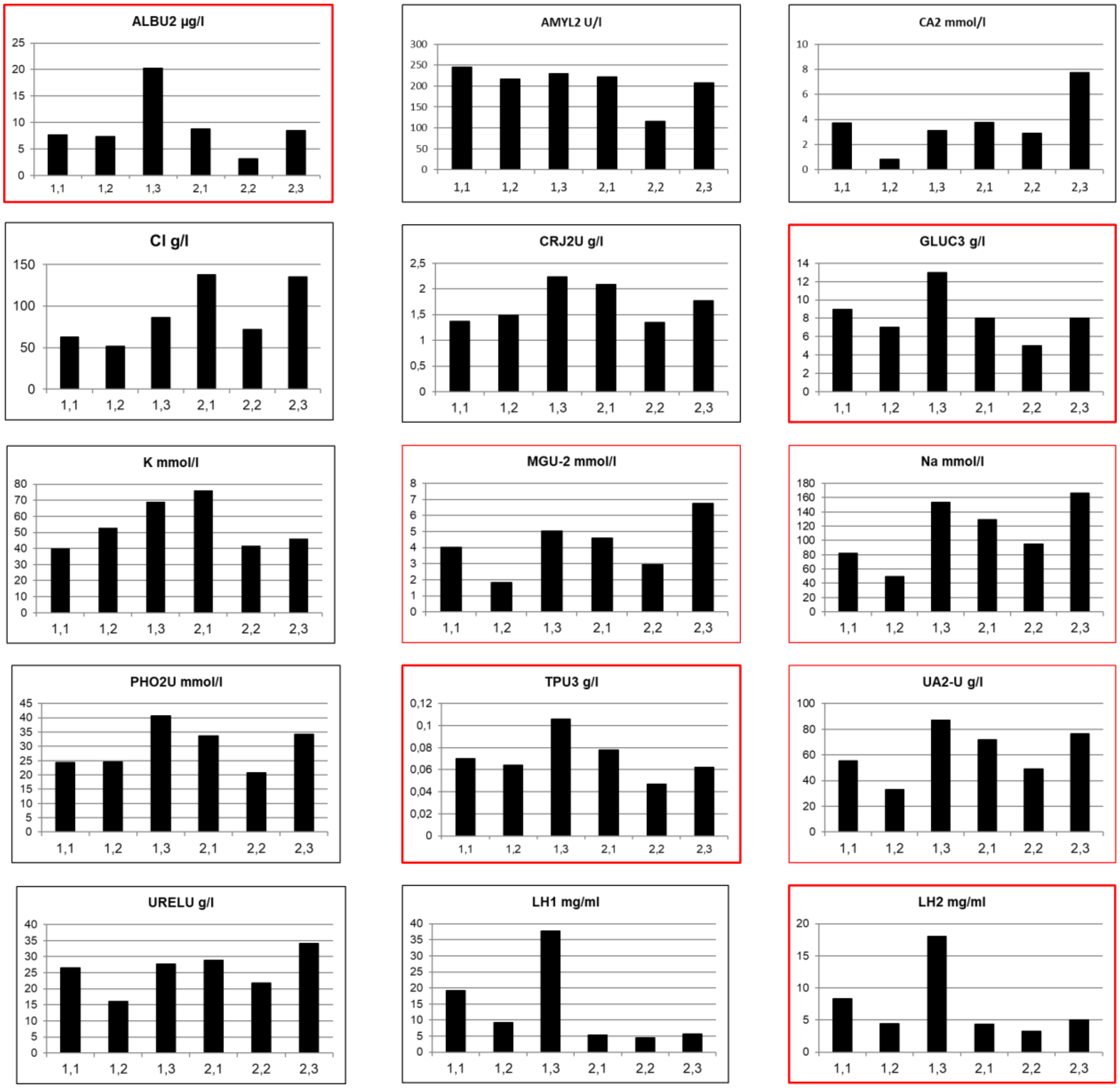

Supplementary Figure 2. Analysis of urine biochemical parameters in longitudinal urine samples. Those that best correlate with the parameters of EV content in respective samples are boarded with red line.

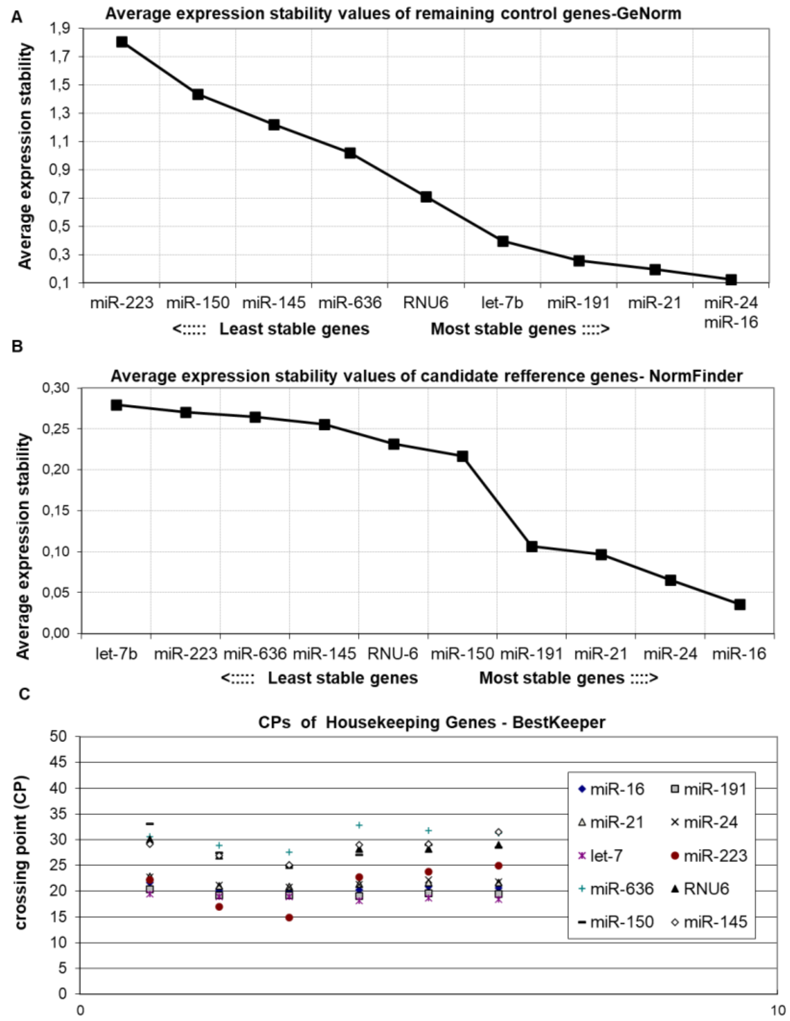

Supplementary Figure 3. Ranking the analyzed EV miRNA as plausible normalizers by calculating their overall expression stability by three statistical algorithms, namely GeNorm, NormFinder and BestKeeper

Figure 2C

UC 1 Month

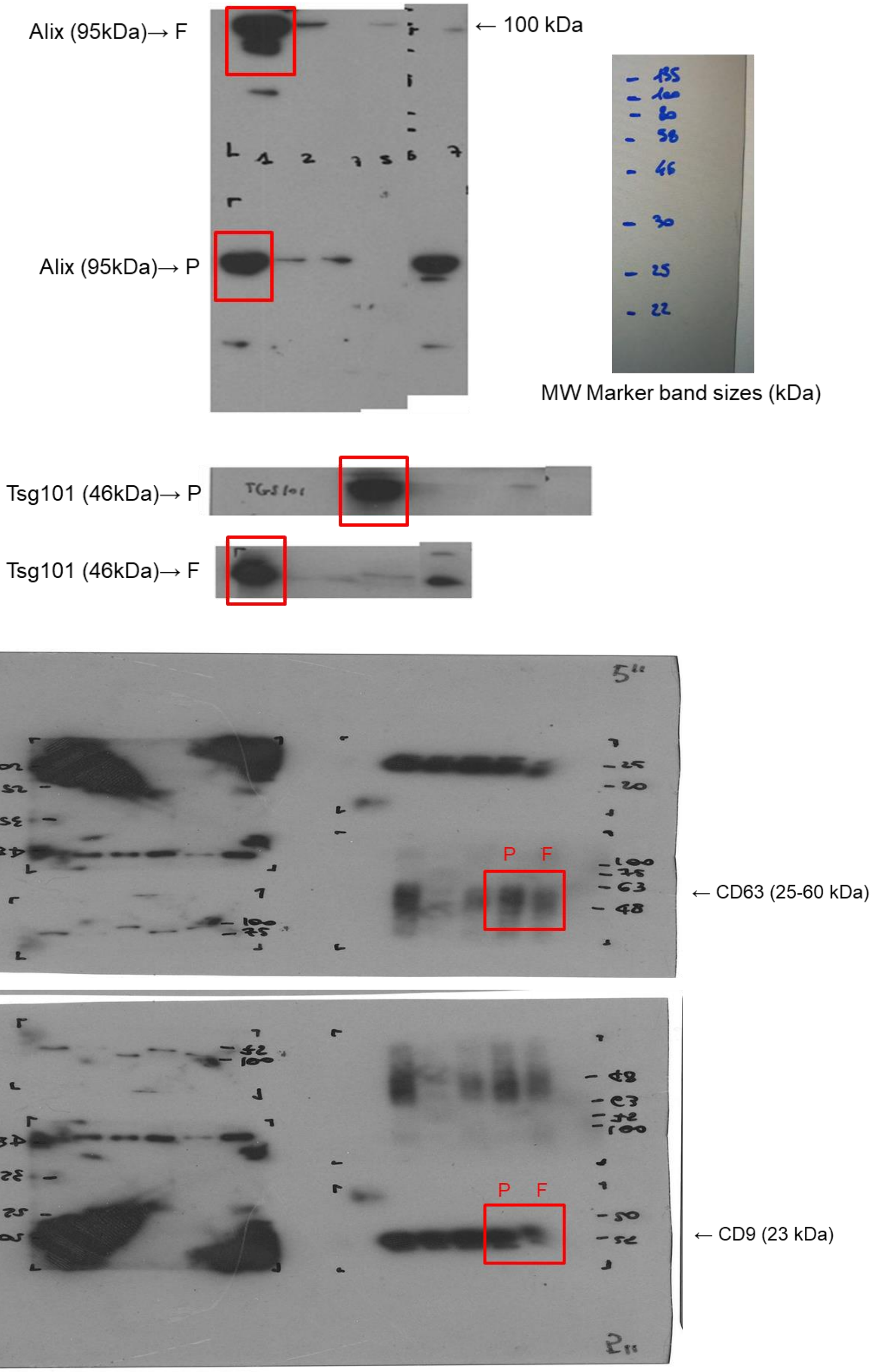

Figure 2C

UC 6 Month

Alix (95kDa)→

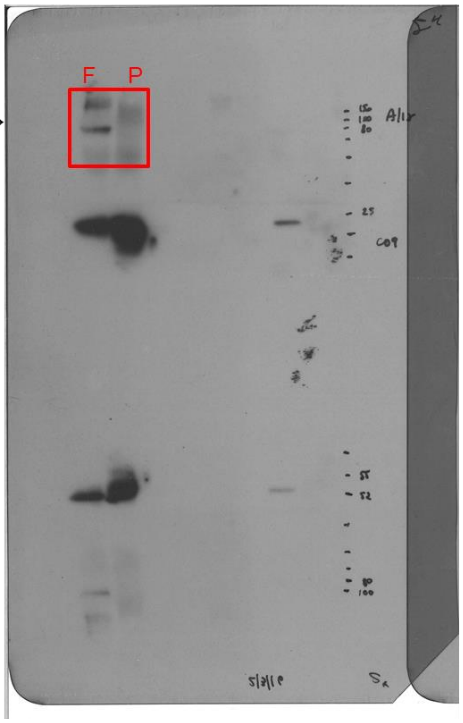

Tsg101 (46kDa)→

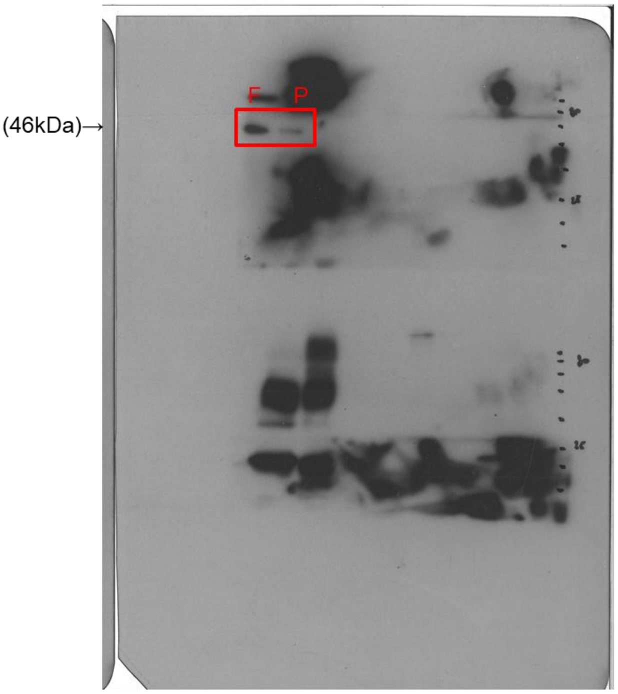

CD63 (25-60 kDa)→

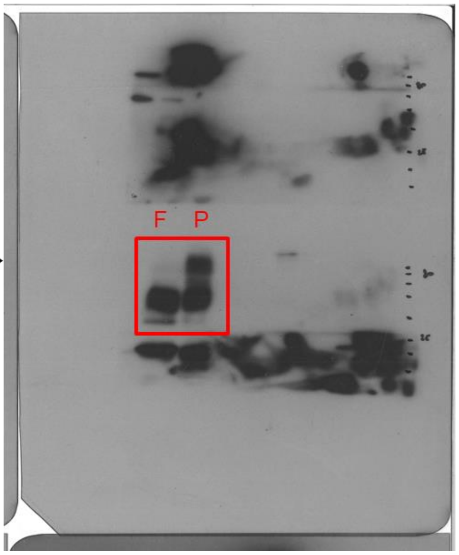

CD9 (23 kDa)→

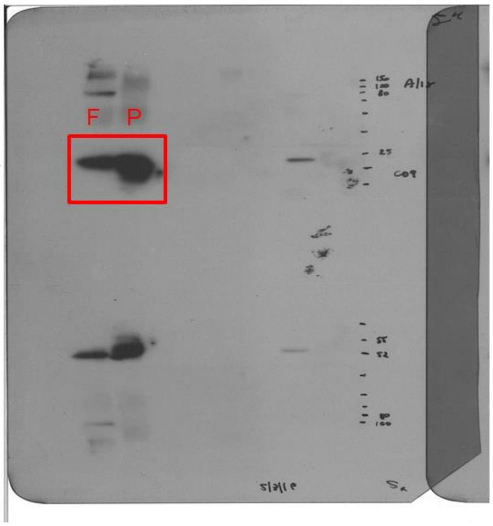

Figure 5C

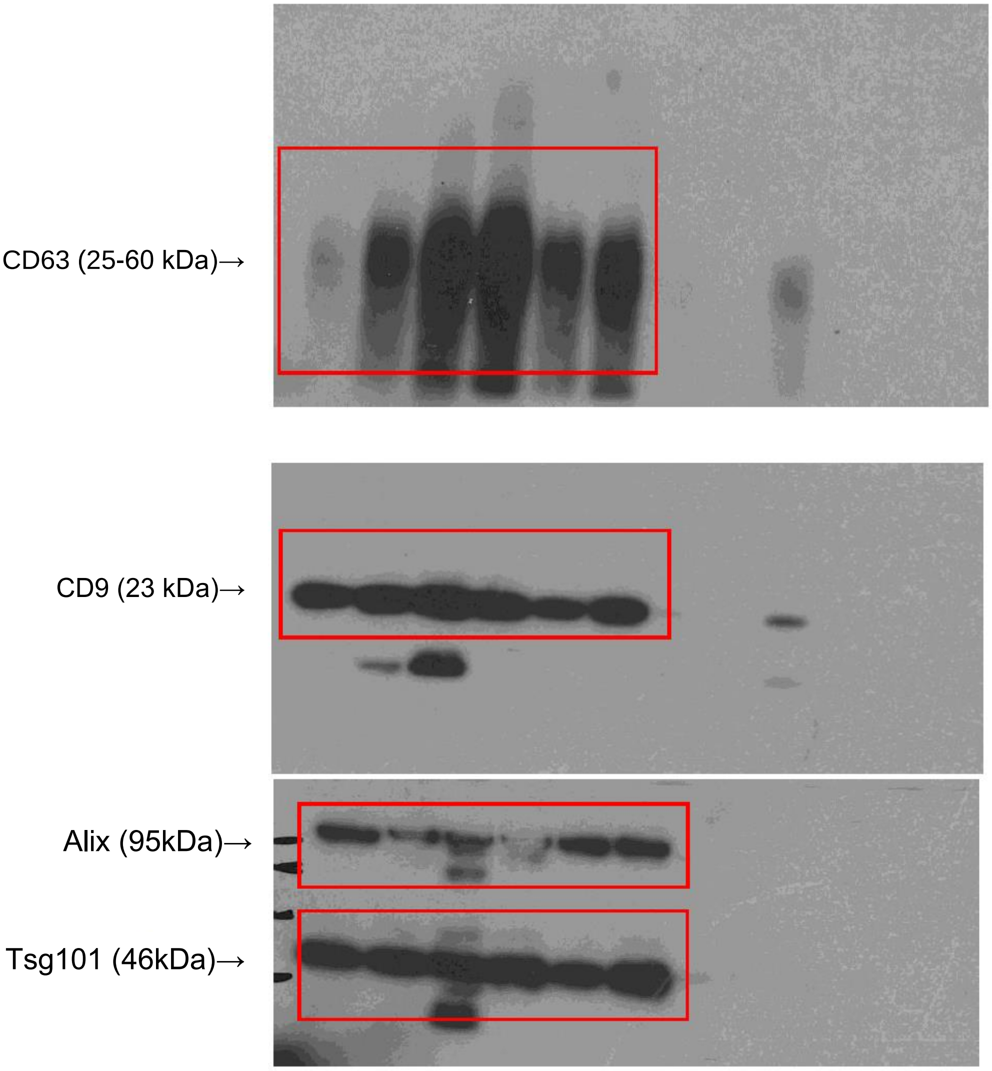

Supplementary Figure 4. Full blot images for figures 2C and 5C. Hereby the original blots for are shown, some of which (including the blot for Tsg101 in Figure 2C, UC 1Month Panel, as well as for figure 5C blots) were cut prior to hybridisation with antibodies during blotting; fuller-length blot performed with other samples using the same antibody is shown in following pictures (See Figure 2C UC 6 Months panel), evidencing that the detected band confirms specific detection (expected MW) of the target antigen. Clearly indicated MW band sizes from used MW marker are also displayed
